# Application of Dynamic and Static Light Scattering for Size and Shape Characterization of Small Extracellular Nanoparticles in Plasma and Ascites of Ovarian Cancer Patients

**DOI:** 10.3390/ijms222312946

**Published:** 2021-11-30

**Authors:** Ksenija Kogej, Darja Božič, Borut Kobal, Maruša Herzog, Katarina Černe

**Affiliations:** 1 Faculty of Chemistry and Chemical Technology, Department of Chemistry and Biochemistry, University of Ljubljana, SI-1000 Ljubljana, Slovenia; darja.bozic@zf.uni-lj.si; 2 Laboratory of Clinical Biophysics, Faculty of Health Sciences, University of Ljubljana, SI-1000 Ljubljana, Slovenia; 3 Division of Gynecology and Obstetrics, Department of Gynecology, University Medical Centre Ljubljana, SI-1000 Ljubljana, Slovenia; borut.kobal@kclj.si (B.K.); marusa.herzog@kclj.si (M.H.); 4 Faculty of Medicine, Department of Gynecology and Obstetrics, University of Ljubljana, SI-1000 Ljubljana, Slovenia; 5 Faculty of Medicine, Department of Pharmacology and Experimental Toxicology, University of Ljubljana, SI-1000 Ljubljana, Slovenia; katarina.cerne@mf.uni-lj.si

**Keywords:** extracellular vesicles, extracellular nanoparticles, dynamic light scattering, static light scattering, particle size, particle shape, ovarian cancer

## Abstract

In parallel to medical treatment of ovarian cancer, methods for the early detection of cancer tumors are being sought. In this contribution, the use of non-invasive static (SLS) and dynamic light scattering (DLS) for the characterization of extracellular nanoparticles (ENPs) in body fluids of advanced serous ovarian cancer (OC) and benign gynecological pathology (BP) patients is demonstrated and critically evaluated. Samples of plasma and ascites (OC patients) or plasma, peritoneal fluid, and peritoneal washing (BP patients) were analyzed. The hydrodynamic radius (*R*_h_) and the radius of gyration (*R*_g_) of ENPs were calculated from the angular dependency of LS intensity for two ENP subpopulations. *R*_h_ and *R*_g_ of the predominant ENP population of OC patients were in the range 20–30 nm (diameter 40–60 nm). In thawed samples, larger particles (*R*_h_ mostly above 100 nm) were detected as well. The shape parameter ρ of both particle populations was around 1, which is typical for spherical particles with mass concentrated on the rim, as in vesicles. The *R*_h_ and *R*_g_ of ENPs in BP patients were larger than in OC patients, with ρ ≈ 1.1–2, implying a more elongated/distorted shape. These results show that SLS and DLS are promising methods for the analysis of morphological features of ENPs and have the potential to discriminate between OC and BP patients. However, further development of the methodology is required.

## 1. Introduction

Ovarian cancer (OC) is a highly aggressive type of tumor. It is the third in terms of incidence of tumors of the female reproductive system and the first in terms of death rates of gynecological malignancies in women, leading to almost 185,000 deaths annually worldwide [1,2,3,4]. Approximately 90% of malignant ovarian tumors are epithelial in origin [3,4], and among them, the serous histological subtype is the most diagnosed and responsible for the most OC deaths [1,5,6]. Although OC patients initially often respond to chemotherapy, they usually develop chemo-resistance at a later stage. Recurrence is frequently accompanied by the development of carcinomatosis, which may not be amenable to surgery [7], and is almost always fatal.

These facts clearly indicate the need for early diagnosis of the disease, which, however, requires new analytical approaches. In this context, extracellular vesicles (EVs), including the recently frequently investigated exosomes, are gaining considerable research interest due to their diagnostic and therapeutic potential, in particular in cancer therapy [8,9,10,11,12]. The release of EVs into human blood and other body fluids (e.g., ascites, urine, and saliva) may be enhanced several times under pathological conditions [13,14,15,16,17].

It was recently shown by the method of measuring and analyzing ionic currents that EVs from particular cancer cells, cultured in Dulbecco’s modified Eagle’s medium, exhibit specific morphologies [18]. The authors argued that the shape distributions of EVs suspended in a solution have the potential to discriminate between cancer cells. Size and shape are, thus, potentially useful information for clinical diagnostics.

Altered lipid metabolism has emerged as another important feature of OC. Recent studies show that circulating lipid profile may be associated with the risk in OC [19,20,21,22]. Therefore, not only EVs, but also lipoproteins may be of interest in the OC prognosis and monitoring of the disease [8,11,12,19,20,21,22]. EVs (sizes from 30 to ~1000 nm [23], which includes exosomes with sizes ~30–120 nm [11,24]), lipoproteins (sizes ~7–600 nm [25]), large protein complexes, and other particles in the overlapping size range, can be present in complex biological samples in different proportions. Focusing on the size, a more general term “extracellular nanoparticles” (ENPs) is used in this contribution to refer to all these particles.

Owing to the submicron size of ENPs, the technique of choice for their characterization is the scattering of visible light. Light scattering (LS) is a highly reliable method for size and shape analysis of nanosized particles. Its major advantage is that it allows for the analysis of complex fluids and is independent of particle type. Due to the non-specificity and high sensitivity, LS is gaining considerable interest in studies of complex colloidal systems, such as blood plasma [26,27]. In this contribution, we explore the possibility to use LS for discrimination between different cancer pathologies [8,11,12], focusing on the small ENP population (<250 nm) in conjunction with a malignant and benign pathology of OC.

Several other techniques were tested in the past for size determination of EVs isolated from blood, for example scanning or transmission electron microscopy (EM) [13,28,29,30] nanoparticle tracking analysis (NTA) [17,31,32,33,34,35], and flow cytometry (FC) [33,34,35]. It was argued [13,29,30] that EM underestimates the size of vesicles; in addition, the real size distribution of the entire vesicle population is not assessable by this method [36]. NTA appears to be more convenient to use, but still has certain drawbacks: for example, two populations can only be resolved if their particle diameters differ at least by a factor 1.5 [37]. The limitation of conventional FC, on the other hand, is the low detection limit of this technique with respect to the particle size (300–800 nm in older and 160–220 nm in newer generation FC [33,34]), which is mostly above the usual size of lipoproteins and exosomes. Atomic force microscopy (AFM) is also appropriate for the sizing of vesicles [26,28], but sampling considers a relatively small number of particles. High-resolution AFM was employed for single vesicle observations of salivary exosomes from oral cancer patients and revealed irregular morphologies and increased vesicle sizes [28]; however, the generalization of results from single vesicle studies to the entire vesicle population is difficult.

New methods, such as asymmetric flow field flow fractionation coupled with multi-angle light scattering (AF4-MALS), tunable resistive pulse sensing (TRPS), and high-sensitivity nano-flow cytometry (nFC), were found suitable for the determination of concentration and number-weighted particle size distributions of heterogeneous populations of EV isolates as well [35]. In the study of Sitar et al. [37], results from AF4-MALS were supported by conventional LS, indicating the power of LS.

The combination of static (SLS) and dynamic light scattering (DLS) presents a powerful approach for the determination of size and topology/morphology of particles in various soft matter systems, most frequently in polymer solutions [38,39,40,41,42,43]. Recently, the method was tested also for lipoproteins [44,45] and various vesicular systems, for example liposomes [46], exosome standards [26,37], or EVs isolated from human blood [26,47]. Research demonstrated [26,37,46,47] that careful analysis of LS data provides important structural information about vesicles in addition to the particle size, such as their size distribution, shape, and mass distribution within the particle, in conjunction with the effect of the isolation procedures on these parameters. Further advantages of LS are that it is not destructive for the analyzed samples and that it allows analysis of the particle size distribution directly in the complex biological fluid, avoiding sample dilution, fractionation or change of the medium, which could affect the presence (or properties) of the assemblies that depend on the system equilibrium. However, special care must be taken in harvesting the material and in analyzing the LS data.

Size distributions in real colloidal systems are often multimodal, i.e., samples can contain more than one clearly identifiable population of particles. Analysis of such multimodal systems presents a major challenge in batch methods, like DLS and SLS. An approach to de-convolute the total intensity of scattered light into individual contributions of two subpopulations was recently suggested in the literature [40,41,42,43,48] and was already successfully tested for the characterization of exosome populations in exosome standards [26,37]. Moreover, Božič et al. [26] performed a critical assessment of this approach on exosomes isolated from blood of healthy donors and have convincingly shown that viscosity of the medium is the critical parameter in accurate size and shape determination of vesicles by LS. As far as our knowledge is concerned, such combined SLS and DLS analysis was until now not undertaken in characterization of EVs in blood samples of OC (or other pathology) patients.

It is also necessary to stress that biological fluids contain various types of supramolecular assemblies. The established methods for EV separation profoundly affect the final composition of the EV “isolates” [49,50,51]. Many nanosized particles (particularly EVs) can be formed by excessive processing of the samples [47,52]. Therefore, minimization of processing is sought as favorable to enable insight into the innate presence of the circulating ENPs in biological fluids. This was the goal pursued in this study.

EVs [53,54] and lipoproteins [55,56] both play roles in the intercellular transfer of material and communication and exhibit many common features [57]. In a majority of studies, EVs and lipoproteins are perceived as strictly separate classes of particles, and represent an interference or “contamination” in the isolates of one-another. Both EVs and lipoproteins, are clearly diverse and heterodisperse groups of phospholipid-coated (bilayer or monolayer, respectively) particles. The separation of specific subgroups can hardly be achieved, and a rigorous assessment is needed to avoid misconceptions in data interpretation [58]. In this investigation, small sized ENPs (<250 nm) in body fluids of patients with OC pathology were analyzed as a population. Structural characterization by multiangle LS assessment, exploiting the de-convolution method, was then implemented for the characterization of subpopulations that were identified based on the obtained size distributions.

We collected and analyzed paired samples of plasma and ascites of OC patients. As a control, plasma, peritoneal fluid (PF), and peritoneal washing (PW) of patients with a benign pathology (BP) of reproductive organs were investigated (see Materials and Methods). Additionally, the influence of various commonly used procedures in the preparation of ENP samples, e.g., filtration, freezing-thawing, storage, and differential ultracentrifugation, on the size and topology of ENPs was evaluated. To our knowledge this is the first such study of the scattering profile of the ENPs in plasma and ascites of patients with OC and BP pathologies. Due to the logistical and other difficulties (acquiring sufficient number of patients in a reasonable time-frame), the number of analyzed cases in this study is relatively small. However, the observed potential to discriminate BP and OC patients makes the presented DLS/SLS approach a promising tool. Further optimization and reevaluation/validation of the method on a larger number of samples is needed in the future.

## 2. Results

### 2.1. Preliminary DLS Measurements at θ = 90°

**Figure 1:**
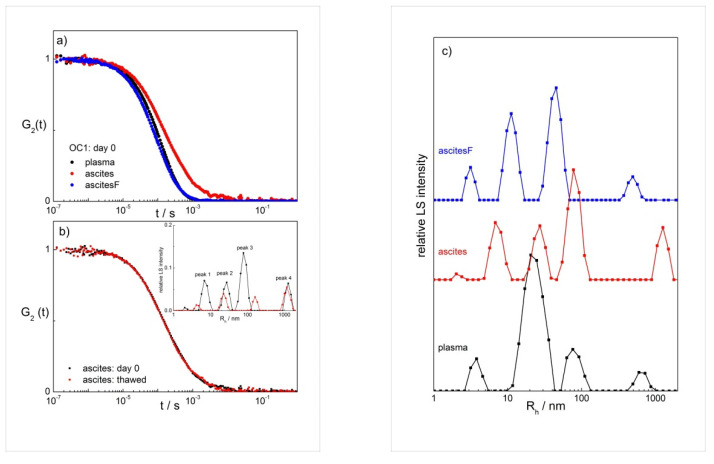
(**a**) The normalized intensity correlation functions (*G*_2_(t)) of plasma, ascites, and filtered ascites (ascitesF) measured on the day of sampling (day 0) at θ = 90° and *T* = 25 °C; (**b**) the effect of freezing/thawing on *G*_2_(t) and the calculated distributions of the hydrodynamic radii (*R*_h_) of particles in ascites (see inset); (**c**) the intensity weighted distributions of the hydrodynamic radii (*R*_h_) of particles in plasma and both ascites samples. All data are for ovarian cancer patient OC1. The distributions are calculated by considering the viscosity of water at 25 °C (*η*_o_ = 0.9 mPa s^−1^).

We start with presenting DLS measurements at a single scattering angle θ (=90°), which is the approach usually followed in DLS studies of ENPs and exosomes in the past [59,60,61]. Correlation functions at θ = 90° were collected for plasma and two ascites samples of patient OC1, unfiltered (designation ‘ascites’) and filtered (designation ‘ascitesF’; see Section 4). Note that filtering is necessary in LS experiments, especially in the case of aqueous systems, which have a large tendency to bind dust particles as these may have a strong effect on the collected LS data. Therefore, the impact of filtering needs to be evaluated. The effect of time after the sampling and of freezing (followed by thawing) of the samples was followed as well (see Figure S2-left panel in Supplementary Material). Examples of the normalized *G*_2_(t) functions measured on the day of sampling (day 0) are shown in Figure 1a, and the calculated size distributions in Figure 1c. The shift of *G*_2_(t) to longer relaxation times, which is the case with ascites (*c.f.* the red curve in Figure 1a), demonstrates the presence of larger particles in this sample. This is seen also from the calculated size distributions for ascites, in comparison with other samples, where peaks at higher *R*_h_ values dominate in the distributions (Figure 1c).

The freezing and thawing of samples caused a decrease in the LS intensity and a simultaneous decrease in the peak heights for all samples. This is shown for ascites in Figure 1b (inset) and for plasma, ascites and ascitesF in Figure S2 (Supplementary Materials; the right panel). The likely cause for these observations is the partial disintegration of particles (ENPs) induced by exposure of samples to temperature shocks. It will be discussed below that simultaneously freezing and thawing led to an increase in the number of particles represented by peak 3.

The study of the effect of time after filtering on the results shows that *G*_2_(*t*) did not depend on time when the measurement was performed (Figure S2, left panel). Only the correlation functions for ascites did not overlap perfectly; however, this could be attributed to the fact that the sample was not filtered. Dust particles could cross the light beam during the measurement and cause a shift of *G*_2_(*t*) to longer times, suggesting that the unfiltered ascites was less stable with respect to time than other samples. The decrease of the LS intensity with increasing time for all samples in Figure S2 indicates that aggregates (large particles) were partially disappearing with time (likely disintegrating; eventual sedimentation is ruled out because the samples were gently turned up and down prior to each measurement). It is, therefore, recommended to perform the LS analysis on fresh and filtered samples, which was the approach followed below. If the analysis is not possible on the day of sampling, ascites samples should be filtered before storage. Freezing of the samples affects the particle distribution in all samples and is, therefore, not recommended.

### 2.2. Analysis of LS Data at θ = 90°

The main purpose of this part is to examine the size distributions obtained by DLS and identify sizes (in terms of the hydrodynamic radius, *R*_h_) of particles that are present in the samples. The mean *R*_h_ values and the calculated size distributions at θ = 90° for the plasma, ascites, and ascitesF of patient OC1 are reported in Table 1 (additional values are collected in Table S3 in Supplementary Materials for all OC samples and all days). Peak 1 normally appears at *R*_h_ below 10 nm and is attributed to various proteins, nucleic acids, etc. that remain in suspension even after ENP isolation by differential ultracentrifugation. The *R*_h_ values of peak 2 were found in the range of 15–35 nm (diameter 30–70 nm) and those of peak 3 above 70 nm (diameter above 140 nm; see bold *R*_h_ values in Table 1, obtained with *η*_o_ ≈ 1.2 mPa s^−1^; the ones obtained with viscosity of water are reported in Supplementary Materials). In agreement with literature reports [11,26,37,62], peaks 2 and 3 are attributed to ENPs, with peak 2 typically assigned to exosomes [11]. However, other types of ENPs can be present in the assessed populations. Peak 4 was observed at rather irregular *R*_h_ values of a few 100 nm to a few 1000 nm, irrespective of filtering the samples immediately before the LS measurement or not. These values are not reported in Table S3 because the laser wavelength (633 nm) prevents their reliable determination. They are attributed to larger species—possibly aggregates of smaller particles.

**Figure 2:**
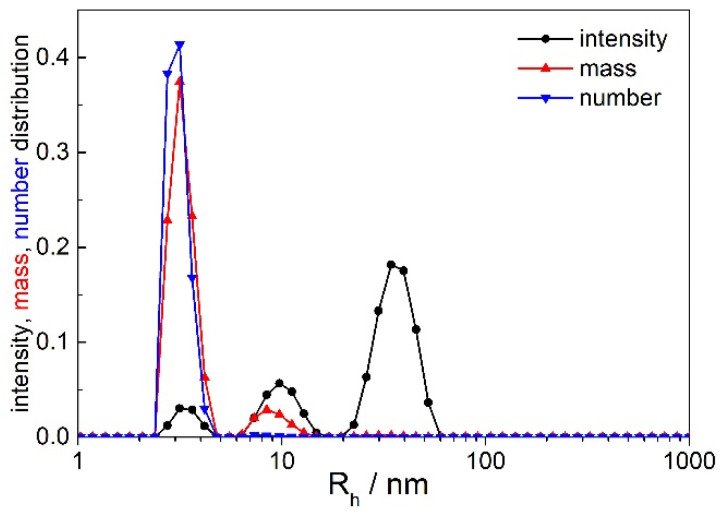
The intensity, mass, and number weighted distributions of particles in the plasma of ovarian cancer patient OC5: θ = 90°, *T* = 25 °C. The distributions were calculated by considering the viscosity of water (*η*_o_ = 0.9 mPa s^−1^).

Peaks 1 and 2 were always observed. The size of the smallest particles (peak 1) was too small to obtain any angular dependency of the scattered light intensity; consequently, these species were not analyzed further. Peak 2 is attributed to the prevailing population of ENPs, whereas peak 3 represents the minority ENP population and was not always observed. In the following, we use the terminology small ENPs (due to their smaller size in comparison with peak 3) and large ENPs [37,62] to label these vesicles. Mostly, the number of the large ENPs was very small; in the number distributions, peak 3 almost disappeared (see an example of intensity, mass, and number distributions in Figure 2). Accurate analysis of angular dependency of the scattered light attributed to this population could be performed only in some cases (patients OC6 and OC7), which will be discussed in detail below. The angular dependency of scattered light was, thus, followed for small ENPs (peak 2) and, when possible, for large ENPs (peak 3).

Figure 2 deserves additional comments. Clearly, large ENPs (peak 3) dominate in the intensity weighted distribution, whereas the smallest particles (peak 1) scatter very weakly and contribute much less to the total LS intensity. This is expected because light scattered, e.g., by a spherical solid (or hollow) particle depends on the sixth (or fourth) power of its diameter. Figure 2 clearly demonstrates that most of the particles in plasma and local fluids of the herein studied patients are those with sizes below 10 nm (proteins, nucleic acids and alike). The example in Figure 2 displays three peaks; larger aggregates (*R*_h_ ≥ 1000 nm) were not identified at θ = 90° in that case.

Similar results as for patient OC1 were collected also for one of the patients from the control group (patient BP-M). The measured correlation functions for plasma, PW and PF of this patient are shown in Figure S3 (Supplementary Materials) for days 0 and 1, but not for longer times because these samples; in particular, PW and PF, were rather unstable. The LS intensity of PW was very low showing that the concentration of particles in PW is low. This is reflected also in the quality of the *G*_2_(*t*) function recorded in this case (see, for example, the stronger fluctuations at short correlation times in Figure S3b). The best result with PW was obtained for the filtered sample, which was, therefore, also repeated on day 1. The peak positions for the plasma, PW, and PF of patient BP-M are reported in Table S4 (Supplementary Materials). Again, we detected peaks in a similar size range as reported for patient OC1: peak 1 (${\bar{R}}_{h}\;$< 10 nm), 2 (${\bar{R}}_{h}$ ≈ 10–30 nm), 3 (${\bar{R}}_{h}$ a few hundred nm), and 4 (${\bar{R}}_{h}$ > 1000 nm; not reported in the tables). The most important result from these preliminary measurements on patients with BP (patient BP-M) is that particles in the size range ${\bar{R}}_{h}$ ≈ 25 nm, which are usually attributed to exosomes, were not detected in fresh PW.

### 2.3. Angular Dependency and the Determination of R_g_ and ρ

**Figure 3:**
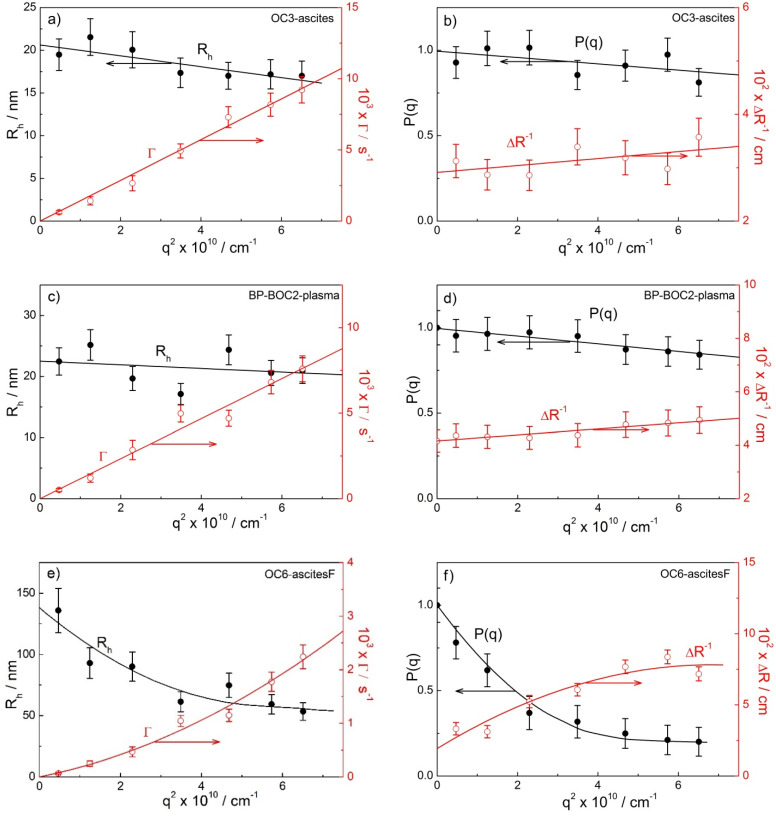
(**a**,**c**,**e**) Hydrodynamic radii (*R*_h_; filled black circles) and decay rates (Γ; empty red circles) and (**b**,**d**,**f**) form factor (*P*(*q*)) and the reciprocal of the excess Rayleigh ratio (Δ*R*^−1^) as a function of the square of the scattering vector (*q*^2^) for small ENPs (peak 2) in the ascites of ovarian cancer (OC) patient OC3 (**a**,**b**) and the plasma of patient with benign ovarian cyst (BP-BOC2) (**c**,**d**), and for large ENPs (peak 3) in the filtered ascites (ascitesF) of patient OC6 (**e**,**f**). In the calculation of *R*_h_ values for large ENPs (Figure 3e), the viscosity value *η*_o_ ≈ 1.2 mPa s^−1^ was considered (see Section 4).

Single-angle measurements do not allow the determination of the radius of gyration (*R*_g_; see Section 4). For this purpose, the angular dependency of the time-averaged LS intensity needs to be analyzed in the cases when the particles are large (fulfilling the criterion *qR*_g_ > 1.5; see Section 4). This is the case for ‘peak 2′ and in some cases for ‘peak 3′ particles, for which the *R*_g_ and the shape parameter ρ (=$R_{g}/R_{h}$) could be evaluated. To calculate ρ, the *R*_h,0_ values (a DLS result) at θ = 0° are determined first. In the case of peak 2, *R*_h_ either decreased linearly with the increasing angle (as examples in Figure 3a,c for the samples OC3-ascites and BP-BOC2-plasma, respectively) or was independent of the angle (see Figure S4a,c in Supplementary Materials for the samples OC4-plasma and BP-BOC2-PF, respectively). For monodisperse spherical particles of any size, it is expected that *R*_h_ is independent of θ (or the scattering vector *q*; see Section 4). However, the polydispersity of species may also have an influence on the angular dependency of *R*_h_, as is clearly demonstrated by the results in Figure 3. The slight decrease of *R*_h_ with increasing *q* agrees with the simulated *R*_h_ values of polydisperse vesicles with an average size of 60 nm [63]. Thus, either the *R*_h_ value extrapolated to θ = 0° (in the case of week angular dependency) or an average *R*_h_ (in the case of constant values) is considered in the evaluation of ρ for small ENPs.

In Figure 3a,c, the decay rate curves (Γ as a function of *q*^2^) are also shown for small ENPs. The dependency is linear in this case, in agreement with the relationship Γ = 1/τ = *Dq*^2^, and the curves pass through the center of coordinate system (through zero), which proves that they represent a true diffusive process of particles [38,39]. This is taken as a proof that the treatment of the DLS data is performed properly and shows that size distribution in the subpopulation of small ENPs is rather narrow.

In the case of ‘peak 3’ particles, the dependency of *R*_h_ on *q*^2^, so as that of Γ on *q*^2^, is curved and the second order polynomial function is used for extrapolation to *q* = 0 (as an example of the extrapolation procedure see Figure 3e). It is important to stress that the Γ vs. *q*^2^ curve passes through zero also in this case and, thus, applies to real translational diffusion of these particles. If this is not the case, such diffusive mode may be a consequence of inter-particle interactions [38,39]. Clearly, this option may be ruled out in the present case. The curvature in the decay rate curve in Figure 3e at higher angles (higher *q*) is attributed to the polydispersity of large ENPs, which is larger in comparison with the small ones (compare the Γ vs. *q*^2^ curves in Figure 3a,c,e). In addition to the DLS data (*R*_h_ and Γ), Figure 3b,d,f also shows examples of the SLS data for the same samples, i.e., the dependency of the form factor *P*(*q*) (calculated from time-averaged LS intensity; *c.f.* Equation (3)) and of the reciprocal absolute LS intensity (Δ*R*^−1^) on *q*^2^ (for details on SLS treatment see Materials and Methods and ref. [64]). The *R*_g_ is calculated from the limiting slope of these lines (*c.f.* Equation (3)). The *P*(*q*) vs. *q*^2^ plots in Figure 3b,d and in Figure S4b (Supplementary Materials) are linear, in agreement with Equation (3), and are always found with small ENPs. Figure 3f and Figure S4d (Supplementary Materials) show examples of curved plots that are fitted with the second order polynomial dependency of *P*(*q*) on *q*^2^ (*c.f.* Equation (S7a); Supplementary Materials). Such dependencies are detected in the case of peak 3 in some OC patients and in some cases for peak 2 in BP-BOC patients, where the ‘peak 2′ ENPs are larger in size (*R*_h,0_ above 35 nm) in comparison with OC patients.

The obtained *R*_g_ values are collected in Table 2 (all OC patients), Table 3 (all BP-BOC patients), Table S5 (patients OC6 and OC7), and Table S6 (BP-E patient), together with the *R*_h_ values at *q* = 0 (denoted as *R*_h,0_) and the resulting ρ (=*R*_g_/*R*_h,0_) values. Note that the *R*_h_ values for particles with diameters above 100 nm, which are given in bold in Table 2 (patients OC6 and OC7), were calculated with the viscosity of the medium set to *η*_o_ ≈ 1.2 mPa s^−1^ (at 25 °C). As discussed in detail in the Materials and Methods, *η*_0_ is the critical parameter in the correct *R*_h_ determination of particles in plasma and ascites, which are rather dense and viscous environments. It was shown recently [26] that an appropriate *η*_0_ value for the *R*_h_ calculation of large vesicles floating in plasma is around 32% higher than that of pure water (for which *η*_o_ = 0.90 mPa s^−1^ at 25 °C). Here, the higher *η*_o_ (≈1.2 mPa s^−1^) is an approximation chosen for both plasma and ascites. This choice is based on a previous analysis of blood plasma [26] and considering a similar total protein content of blood plasma and ascetic fluids in OC patients as reported in the literature (60–80 mg/mL, typically taken as a serum reference [65], and 81.79 ± 10.40 mg/mL, reported for the ascetic fluid of OC patients [66]). A similar high viscosity and correlation between the viscosity and the total protein content of ascitic fluid was reported for samples of ascites with a serum-ascites albumin gradient ≤11g/L [67]. Finally, taking the *η*_o_ of pure water into account in the *R*_h_ calculation would produce far too high results for *R*_h_. To demonstrate this, the *R*_h_ values calculated with the viscosity of water are reported for the same patients (OC6 and OC7) in Table S5 (out of parentheses) and compared with the corrected ones (in parentheses). These are clearly strongly overestimated.

In addition to data derived from the angular dependency of scattered light intensity, Table 2, Table 3, Table S5, and Table S6 also report the mean hydrodynamic radii of the peaks in the size distributions calculated at θ = 90° (*R*_h,90_) and, therefore, also include preliminary data for patients OC1 and BP-M, for which data were collected only at 90°. This is the size that is routinely measured by DLS and is reported in the majority of the previous publications on EV or exosome analysis by DLS. However, as clearly illustrated by examples of *R*_h_ dependency on angle (or *q*^2^) in Figure 3, the *R*_h,90_ values often underestimate the size of particles.

The last column in Table 2, Table 3, Table S5, and Table S6 gives the contribution of the treated particles (either peak 2 or peak 3 particles) to the total intensity of light scattered at θ = 90°. As we can clearly see, smaller ENPs (peak 2) dominate in the intensity weighted distributions in all body fluids of OC patients and in the plasma of BP patients. Their contribution to *I*_tot,90_ is mostly significantly larger than 50% (up to 90%). Large vesicles contribute considerably less. In those OC samples, where this population could be analyzed, their contribution to *I*_tot,90°_ was only a little above 10%. By taking into account that large particles scatter light much more strongly than the small ones (see Section 4 and refs. [38,64]), we conclude that small ENPs dominate over the large ones, which is clearly demonstrated by the number distribution reported in Figure 2.

The mean *R*_h_ values for small ENPs of OC patients determined at 90° (peak 2 in Table 2) are in the range *R*_h,90_ = 15–30 nm, in agreement with preliminary measurements (for comparison see other data for patient OC1 reported in Table S3). The extrapolation to θ = 0° results in less scattered *R*_h_ values in most cases: *R*_h,0_ falls in the range 20–28 nm. An exception is patient OC5 where higher *R*_h,0_ values were obtained (*R*_h,0_ = 34 nm in plasma and ascites and 47 nm in ascitesF). It must be noted that this patient was diagnosed with high-grade serous carcinoma of tubal origin. This might be an indication that patient’s pathology influences the EV size. However, to make any further conclusions regarding this result, the body fluids of more patients with the same pathology would have to be analyzed, which is extremely difficult to accomplish. Other (person-related) factors may also have an impact on the vesicle sizes.

The calculated *R*_g_ values are comparable with the *R*_h_ ones, which, for most cases, leads to ρ in the range from 0.97 to 1.16 (average ρ ≈ 1; note that normally around 10% uncertainty is allowed in the determination of ρ). In the three cases in our study, ρ was equal to or higher than 1.5 (see the peak 2 values reported in parenthesis in Table 2). Higher values are always observed with thawed samples and may be attributed partly to freezing and thawing, which might deform or destroy the vesicles, and partly to experimental uncertainties and difficulties in data treatment. One must bear in mind that these are ‘real’ samples, not vesicle suspensions prepared in laboratory from well-characterized compounds and in suspensions with known compositions. The composition and history of ‘real’ samples might have an influence on the quality of the results, despite all the precautions in handling the samples, which were followed in our study. Finally, we conclude that the obtained ρ values (ρ ≈ 1) for small ENPs in the body fluids of OC patients indicate that these ENPs have a roughly spherical shape with a higher mass on the rim/surface (lipid bilayer) and a lower one in the interior (aqueous basin), in agreement with the calculated ρ for a hollow sphere (ρ = 1). This is, therefore, the anticipated shape for OC-derived vesicles.

Similar data as for small ENPs could be extracted for large ENPs only for six samples related to patients OC6 and OC7. Five out of those six apply to thawed samples and only one to a fresh sample that was characterized immediately after the operation (*c.f.* the filtered ascitesF of patient OC6). The difference between the *R*_h,0_ (~100–200 nm) and *R*_h,90°_ (~60–170 nm) values of these ENPs is large due to the strong angular dependency of *R*_h_ in this case (*c.f.* Figure 3e and the comment on the effect of vesicle polydispersity on the angular dependency of *R*_h_ above). Together with the calculated *R*_g_ (=115–230 nm), these *R*_h,0_ values resulted in ρ values in the range of 0.99–1.14. Therefore, as with small ENPs, the deviation of ρ from 1 is within the limits of experimental error usually accepted for LS measurements (i.e., ~10%). We thus conclude that the mass distribution in large ENPs is approximately the same as in small ENPs, i.e., they are spherical particles mostly filled with water, but some small amount of low molecular compounds (simple salts and sugars, small proteins) is allowed in their interior as well. Clearly, one would come to a different (incorrect) conclusion by considering the viscosity of water when calculating *R*_h_. The resulting ρ values in that case would be lower (ρ = 0.75–0.88; see Table S5), suggesting that particles have a roughly uniform density throughout, as is the case with a hard sphere (ρ = 0.78 [38] and equations in Supplementary Materials). Our results, thus, show that great care must be taken when evaluating the size and shape of ENPs from DLS measurements in different media. Ignoring the medium viscosity (considering the water viscosity in all cases, as is usually the case in literature reports) leads to overestimated *R*_h_ and underestimated ρ values.

These results in ρ are the first ones obtained for ovarian cancer patients; therefore, direct comparison with literature data is not possible. However, a similar ρ was reported for small platelet-derived ENPs in ref. [68]. In that study, ρ = 0.95 was obtained for vesicles with *R*_h_ (=68.5 nm) and *R*_g_ (=64.9 nm). Although the herein studied ENPs may be a heterogeneous group of particles, ρ ≈ 1 suggests that the majority of particles in OC patients are vesicle-like.

In comparison with the LS results for OC patients, the data for BP patients are much more scattered. The *R*_h,90_ is from ~13 nm to more than 40 nm (*c.f.* Table 3 and Table S6), similarly *R*_h,0_ spans from ~10 nm to ~50 nm (20–30 nm in plasma). Although the ‘peak 2′ particles are sufficiently large to expect angular dependency of the LS intensity, very often *R*_g_ could not be obtained for BP patients because the slope of the *P*(*q*) vs. *q*^2^ lines was close to zero (note that *R*_g_ is determined from this slope). This was the case with plasma samples of patients BP-BOC1, BP-BOC3, and BP-BOC4 (Table 3). The *R*_g_ values that are reported in Table 3 and Table S6 are from 25 to almost 70 nm, leading to ρ values larger than 1 (ρ = 1.1–2). The contribution to *I*_tot,90°_ is large (60–90%) for particles with *R*_h_ below 30 nm (found in plasma) and around 20–25% for particles with *R*_h_ above 30 nm (found in PF). This is expected due to the substantial amount of lipoproteins in blood plasma, normally exceeding the number of EVs (~10^16^/mL vs. ~10^10^/mL, respectively [69], among which, small lipoprotein particles prevail in lean healthy subjects in the fasting state [70]). The differentiation between small and large ENPs population is not possible for BP patients. It seems that ‘small’ ENPs found in PF are, on average, larger (*R*_g_ is from 50 to ~70 nm) and with higher ρ values than those found in plasma. The explanation may be that PF is a more dilute suspension of ENPs, and dilution often leads to aggregate growth or possibly fusion in the case of vesicles. Thus, we presume that, in dilute suspensions, the shapes of these aggregates may be more irregular: for example, slightly elongated or distorted in some other way. It should also be noted that, even in a more concentrated vesicle suspension of plasma, the analysis of vesicles for BP patient was often hampered; only two plasma isolates could be analyzed for BP-BOC patients.

An even more dilute fluid, and thus more problematic for SLS/DLS analysis, is PW. However, the advantage is that PW can always be obtained, even when PF is absent. Therefore, we analyzed an additional sample of PW from patient BP-E (see Table S6; sample designated as PW*). The LS intensity of PW* from patient BP-E was comparable to the preliminary results for PW from patient BP-M, except that the particles in the size range corresponding to ‘peak 2′ ENPs were detected in the thawed PW* of patient BP-E (*c.f. R*_h,90_ = 27 nm) but not in the fresh PW of patient BP-M.

To concentrate these dilute ENP suspensions for eventual easier (or more accurate) analysis, we performed differential ultracentrifugation, which is one of the main procedures for the isolation and concentration of EVs. The effect of this pre-analytical step on the contribution of small ENPs to *I*_tot,90°_ was analyzed by comparing the results of ultracentrifuged and non-ultracentrifuged thawed PF* and PW* samples of patient BP-E (Table S6: the ultracentrifuged samples were designated with C or C+F in the case that they were also filtered prior to LS measurement). In contrast to our expectations, the relative contribution of small ENPs to *I*_tot,90°_ for PW* in BP-E after ultracentrifugation was nearly unchanged (compare the values for PW* and PW*-C). On the other hand, additional filtering of samples before the LS measurement increased the contribution of ‘peak 2′ ENPs to *I*_tot,90°_ in both types of local fluids (compare PW*-C with PW*-C+F and PF*-C with PF*-C+F), presumably because strongly scattering larger species (e.g., aggregates) were removed from the suspension by filtration. Additionally, we observed that ultracentrifugation/filtration also led to a pronounced increase in *R*_h_ values: *R*_h,90_ increased from 27 (15) nm for PW* (PF*) to 35 (26) nm for PW*-C (PF*-C) and 75 (86) nm for PW*-C+F (PF*-C+F), respectively, all for patient BP-E; *c.f.*, Table S6). We conclude that the concentration of local fluids by ultracentrifugation may have a considerable effect on the particle size and size distribution, in particular in the case of low viscosity media, such as PW and PF. Our results indicate that the exposure of ENPs to strong centrifugation field resulted in larger sizes. Such growth may also include re-distribution of the material.

## 3. Discussion

Light scattering techniques are used routinely in the field of colloid science for the determination of particle sizes, size distributions, and particle topology. They are known as absolute (no calibration or standard is needed), non-specific, and very accurate techniques, providing that some properties of the system (e.g., correct medium viscosity in DLS) are known. However, LS measurements are mostly conducted on well-defined (with respect to composition) and easily controllable systems. From this point of view, a completely different challenge is the LS characterization of ENPs in body fluids from patients with OC, which was undertaken in this investigation, as the variety of physiological conditions in this case was impossible to control. However, by taking great care in sample preparation and proper analysis of LS data we demonstrated that the combined DLS/SLS technique can provide valuable structural information about ENPs in such non-trivial systems. We emphasize in particular that only LS measurements at several scattering angles can give complete and reliable information on the particle size and (average) topology. This task, however, is rarely undertaken in EV research.

In recent investigations, the size distributions of EVs from various cell lines were studied by A4F-MALS [26,37,59] and also with the DLS/SLS approach, such as in the present case (taking into account angular dependency data) [26,37]. Sitar et al. [37] and Božič et al. [26] identified two subpopulations of exosomes in lyophilized exosome standards, whereas Agarwal et al. [59] obtained a broad but monomodal distribution of exosomes in culture media of human thyroid carcinoma cell lines. Two EV populations were found also in isolates from the blood of healthy donors [26,47]. Furthermore, Gercel-Taylor et al. [17] compared NTA and DLS for the objective definition of size distributions of cell-derived vesicles from ovarian cancer patients, but no angular dependency was followed in that case. A general consideration of the whole population of the small ENPs harvested from blood of patients diagnosed with an advanced stage carcinoma of uterus and evaluation of the characteristic scattering properties of the resolved subpopulations presents a significant step forward. Although not a routine analysis, this combined DLS/SLS approach offers a possibility to be used as a prognostic marker. A further detailed study of the origin of the observed distinguishable scattering profile, validation of the significance and optimization of the method to allow high-throughput of samples, and suitable supporting methods (e.g., viscosity determination) will be needed in the future.

Comparison between paired samples (plasma and ascites) from OC patients provided consistent results in terms of their hydrodynamic radii and shape as well as in terms of their contribution to the total LS intensity. On the other hand, the results for the control group (the BP patients) were much more heterogeneous, which could be due to the variety of benign gynecological conditions in this group. The average size of ENPs in plasma of BP patients was in the same range as for OC patients, and their contribution to the total LS intensity was also sufficient, but the analysis of *R*_g_ was mostly not possible, and consequently the shape parameter for most of the BP samples could not be obtained.

One of the possible reasons for this finding may be a less extensive small EVs production in benign compared to malignant conditions. This interpretation agrees with the study of Gercel-Taylor et al. [17] These authors reported an approximately four-fold increase in the total vesicle concentration in the serum of OC patients in comparison with patients with benign ovarian cysts. Another possible reason could be the overlapping of EV sizes (which seem to have considerably broader distributions) with other particles in the isolates of BP patients. In conclusion, the finding that angular dependency analysis is not possible (or is very difficult) for BP patients offers a possibility to distinguish between OC and BP pathologies.

Furthermore, we compared the sizes of ENPs originating from paired PF and plasma samples of BP patients. PF-derived ENPs are, on average, larger than those found in plasma and, as hypothesized above, have broader size distributions. PF is taken from *cavum Douglasi* and represents local fluid for EV analysis in BP patients, whereas in advanced OC patients, ascites is usually present and is the one analyzed. Comparison of ENPs from both local fluids shows that PF-derived ENPs are larger than the ascites-derived ones. In agreement with our results, a larger size of EVs from normal human ovarian epithelial cell lines (HOSPiC) in comparison with three OC epithelial cell lines (OVCAR3, IGROV1, and ES-2) was reported in ref. [71]. Another explanation for the larger sizes of ENPs in PF (a much more dilute suspension in comparison with plasma or ascites) may be a direct concentration/mass effect. Often, dilution leads to growth of the aggregates that are formed through self-assembly of components (note that lipid bilayers in vesicles are self-assembled structures). Depending on the driving forces in such self-association, the resulting particles may have either narrow (e.g., micelles) or broad size distributions (e.g., laboratory prepared vesicles in the case that lipid components are left to self-organize into bilayers without some external control over their size, such as extrusion). We also presume that certain subtypes of ENPs can exhibit ellipsoidal or elongated shapes, which is reflected in the value of the shape parameter ρ, which is larger than 1, and is also confirmed by EM images in the literature [47,72,73].

A very important result of this study is that both smaller and larger ENPs in the plasma and ascites of OC patients are roughly spherical and have a less dense interior as indicated by the value of the shape parameter ρ, which is, on average, around 1 in both cases. This result was obtained by considering the directly measured viscosity of the plasma medium in *R*_h_ evaluation of large ENPs (for details on the effect of *η*_0_ see ref. [26] and the Section 4). By considering the viscosity of water, as usually applied in biological samples, we would come to a different conclusion regarding the mass distribution within the large ENPs. In this case ρ would be close to ~0.78, which would indicate a homogeneous mass distribution through the whole vesicle volume, such as in a hard sphere. If this population of ENPs also includes lipoproteins, in addition to various vesicles, a shift of ρ to values below 1 would be expected, as the mass distribution within lipoproteins is more homogeneous. However, if the shape of lipoproteins is not perfectly spherical—for example, more elongated, this would again contribute to a higher ρ (>1). Thus, it is very likely that the determined ρ is some average over all the particles that make up the particular ENP population. Additionally, it needs to be stressed that, even in single angle DLS measurements, the use of the correct viscosity value is important, as simply using the *η*_0_ of water may lead to large differences in the obtained *R*_h_ values. In the present case, *R*_h_ is overestimated by more than 30% if we do not consider the actual viscosity. This point is particularly important when using commercial DLS instruments with a preset viscosity value. From this perspective, a revision of the literature data would be needed, as authors frequently do not pay attention to this issue. Another ambiguity in the literature is whether the reported DLS size of ENPs refers to their hydrodynamic radius or diameter.

The average hydrodynamic radius of small vesicles in OC patients is ~25 nm (diameter ~50 nm), which agrees with the literature reports. In previous studies, where exosomes from plasma and ascites of OC patients were analyzed using scanning or transmission electron microscopy, sizes in the range from 30 to 100 nm were reported [13,29,30]. Similar exosome sizes (mean diameter 45–60 nm) as in our study were also reported for three epithelial OC cell lines (OVCAR3, IGROV1, and ES-2; method transmission electron microscopy [32]) and for an exosome standard (*D*_h_ ~50 nm; method AF4/DLS [37]), whereas somewhat larger ones are found for exosomes from culture media (*D*_h_ between 60 and 80 nm; method AF4/MALS [59]). Interestingly, the average ENP sizes of patient OC5 (*D*_h_ between 70 and 100 nm) with high grade serous carcinoma of tubal origin are close to the data reported in ref. [59].

The average size of large ENPs in ascites of some OC patients (*R*_h,0_ ~ 60–170 nm; *c.f.* Table 2) also agrees with literature data for EVs. In an exosome standard, particles with *R*_h_ ~135 nm were identified [37]. Traditionally, exosome sizes are measured by electron microscopy (EM), which gives approximate values for the diameter of these particles in the range from 30 to 120 nm. The problem with EM analysis is that it is typically an ex situ technique and, thus, prone to user bias and to shrinkage of particles during sample preparation, which leads to an underestimation of size [17]. In addition, as highlighted in the introduction, the real size distribution of the entire vesicle population is not assessable by EM [32,71]. EM procedures (fast freezing) are also at odds with our aim of avoiding excessive sample treatment. On the other hand, the difficulty of LS analysis of multimodal samples is that, although the intensity of light scattered by large particles may be considerable, their number in suspension is usually rather small (see Figure 2), which often results in an overestimation of their size. In the herein investigated samples, where large ENPs could be analyzed, their contribution to *I*_tot,90°_ is only a little above 10% (*c.f.* Table 2). This was mostly samples exposed to temperature shocks, which may cause the disintegration of ENPs followed by their partial recurrence. When a deeply frozen sample is brought back to room temperature, the membrane components reorganize into bilayers again and the resulting size of vesicles may be different (larger) than their initial size.

The finding that two ENPs subpopulations of different sizes are present in OC samples agrees with the published study of Colombo et al. [62] where even three subpopulations of exosomes secreted by human dendritic cells (size ranges <50 nm, 50–100 nm, and 100–200 nm as determined by imuno-EM) were reported. The exosome size was associated with different proteins (HRS, STAM1, and TSG101) of the ESCRT machinery involved in exosomes biogenesis, composition, and secretion. As discussed by the authors [62], heterogeneity in the exosome size and their cargo could be attributed to different ESCRT components present in tumor cell lines and in primary cells.

In comparison to OC patients, a differentiation between smaller and larger ENPs population is not possible for BP patients, neither in plasma nor in local fluids, even though particles in the size range similar to ENPs in OC patients are always detected. Analysis of particles in PW gives inconsistent results, which might be attributed to different pathology of reproductive organs. Small ENPs are present in PW obtained from the patient with endometriosis (BP-E) but are absent in fresh PW obtained from the patient with myoma of the uterus (BP-M). Further research on more samples is needed to elucidate adequacy of PW for ENPs analysis. It would be especially interesting to evaluate ENPs in PW from OC patients diagnosed with an early-stage disease, where ascites are absent in 83% of these patients [74], and thus PW represents the only control of local environment in such situation. All this was out of the scope of the present investigation.

**Figure 4:**
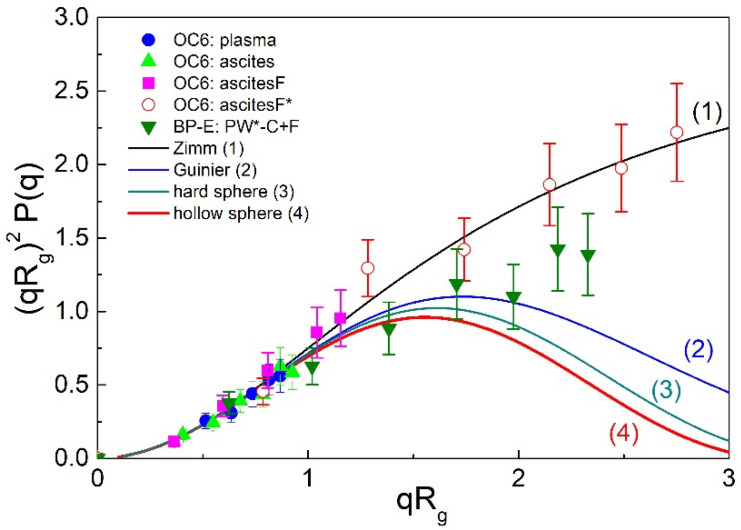
The Kratky plot for four selected particle topologies (solid lines 1–4; for details see Equation (S7a–d) in Supplementary Materials and ref. [38]) and the experimental data for ovarian cancer (OC) patients OC5 and OC6 (the full circles, squares, and triangles apply to small ENPs and the open circles to large ENPs) and for the patient with endometriosis as benign pathology (BP-E) (full down triangles).

In the following, we would like to further validate the above approach for the evaluation of EV shape. The spherical shape is assessed by the value of the shape parameter ρ. In the local fluids of OC patients, it was found to be approximately 1, which is characteristic for vesicles. In our study, ρ was derived from *R*_h,0_ (a DLS results) and *R*_g_ (an SLS results). On the other hand, the SLS results themselves (angular dependency of LS intensity) can also be used for shape evaluation. For this purpose, the form factor *P*(*q*) (*c.f.* Equations (3) and (S7a–d)) is calculated and presented in the form of a Kratky plot (i.e., as a plot of the product ${\left(qR_{g}\right)}^{2}P\left(q\right)$ as a function of $qR_{g}$, or so called scattering function [38]). The experimental data are then compared with calculated form factors for typical particle shapes [38,64]. The Kratky plot is shown in Figure 4 for the body fluids of patients OC5 and OC6 and patient BP-E together with the calculated *P*(*q*) dependencies for some typical particle topologies: the Zimm and Guinier scattering functions are shown together with the *P*(*q*) for a hard and a hollow sphere (for details on these scattering functions see ref. [38,64] and Equation (S7) in Supplementary Materials). The Zimm function is universal and applies to any structure if the product $qR_{g}$ is below 1.2 (signifying that particles are small in comparison with the wavelength of incident light), whereas the Guinier function applies to globular structures with spherical distribution of points around the center of gravity. It is clear from Figure 4 that differences in particle topology become evident only at high $qR_{g}$ values (i.e., when $qR_{g}$ > 1.2).

The data points for plasma, ascites, and ascitesF of patient OC5 in Figure 4 apply to the population of smaller ENPs and are all in the $qR_{g}$ region below 1.2, where differences in particle architecture are not discernible. On the other hand, the data points for the population of larger ENPs from the thawed ascitesF* of patient OC6 extend to larger $qR_{g}$ values (up to $qR_{g}\approx 3$) and follow the Zimm scattering function. The ρ value for this sample (ρ = 0.99) agrees with the theoretical one for a hollow sphere. Similarly, the SLS data for relatively large particles in the thawed sample PW*-C+F of patient BP-E extend up to $qR_{g}\approx 2.5$, but the ρ value is higher (ρ ~ 1.3) in this case. The data points at high $qR_{g}$ values are clearly lower than those for OC6-ascitesF*; they fall below the Zimm function and approach the Guinier approximation, but still deviate from the form factor of a hollow sphere. This Kratky analysis shows that the data points for ENPs from our study do not follow the form factor of a hollow sphere, which is the most suitable shape approximate of vesicles. They also deviate from the form factor of a hard sphere, which could be expected for the lipoprotein particles, as highlighted above.

Two facts need to be emphasized here: (1) the uncertainty in these data points is estimated at around 20% (see the error bars in Figure 4) and (2) ENPs in body fluids are derived from many different cell types, and they are certainly very polydisperse heterogeneous (including protein complexes, exosomes, other EVs, and lipoproteins), which may strongly affect the data at high *qR*_g_ values and complicate the Kratky analysis. Calculations of the form factor of vesicles presented by Pencer and Hallett [63] show that polydispersity contributes to strong positive deviations from the form factor of monodisperse hollow spheres in the Kratky plot at large *q* (or $qR_{g}$) values, in line with the observations in our study. It was also argued [63] that the Guinier approach for the determination of *R*_g_ tends to overestimate the particle sizes in the case of small polydispersities and underestimate in the case of large polydispersities. In agreement with this, Božič et al. [26] determined large positive deviations in the Kratky plot for ENPs in isolates of blood plasma from healthy donors and discussed that eventual aggregation of particles can have a significant contribution to this feature as well.

A detailed study of all these effects is practically impossible for the ENPs from various body fluids in this study and was therefore not undertaken. It would require careful fractionation of particles (along with the determination of composition of separate fractions) and determination of their size distributions also by some other independent and reliable method. One must be aware that any excessive processing necessary in isolation procedures would affect both particle size and topology. Although Kratky analysis is often used in structural characterization of nanoparticles, its use for the analysis of ENPs in blood plasma is, thus, limited.

In conclusion, despite the current lack of understanding of the exact cause of the observed differences between the OC and BP patients, our study illustrates the potential of DLS/SLS in direct analysis of minimally treated complex biological samples. Validation of the observed trends should be performed with appropriate statistics (enough patients), which would require considerably more time and was unfortunately out of the scope of the present study.

## 4. Materials and Methods

### 4.1. Patients

The study included seven patients with advanced OC [FIGO IIIC-IV] and 7 patients with BP of reproductive organs as a control group (see the list of patients in Table S1 in Supplementary Materials). The study was approved by the Commission of the Republic of Slovenia for Medical Ethics (No. 144/12/14) and was in accordance with the Helsinki Declaration. The control group consisted of patients with benign gynecological indications for surgery. The most common indication was benign ovarian cyst (BOC) (five patients with designation BP-BOC).

In addition, one patient with myoma of the uterus (BP-M) and one with endometriosis (BP-E) were considered. Patients were in different stages of their reproductive life. Family, general, gynecological, and obstetric history, indication for surgery, other relevant diseases, and current therapy were collected from medical records. Early-stage cancer (FIGO I, II) and absence of ascites were the exclusion criteria for patients of the OC group. For the control group, a history of malignant disease was an exclusion criterion, and patients with absent PF at laparoscopy or hysteroscopy before the procedure were ruled out. Other details about patients are given in Supplementary Materials.

### 4.2. Collection of Samples

Venous blood samples for the LS analysis were obtained prior to surgery while the patients were hospitalized for preoperative preparation. We drew 4 mL of peripheral blood into vacutainers^TM^ with trisodium citrate after discharging of the first several milliliters because of the platelets activating effect of pressure and contamination by fibroblast [75]. Blood for the analysis of inflammation markers (C-reactive protein (CRP) total white blood cells (WBC)) and OC-standard tumor marker carbohydrate antigene-125 (CA-125) (see Table S1, Supplementary Materials) was obtained together with the samples for ENP analysis.

In patients with OC, ascites was aspirated immediately after the entry into the abdominal cavity using a 50 mL syringe. Bloody samples were discarded, and those patients were excluded from the study. In the control patients, samples of PF and PW were collected during laparoscopy. Immediately after entering the abdominal cavity, all the available PF was aspirated from the *cavum Douglasi* using a syringe through an accessory trocar. This was followed by the washing procedure using 20 mL of Lactated Ringer’s solution.

In our previous study [76], we standardized the sampling protocol for PF and PW to ensure reliable results. We standardized the main factors of the sampling procedure that can affect the tumor marker concentration: the solution volume and the time the solution is left in the pelvic cavity, specification of areas for washing and accuracy during aspiration of the whole solution volume (in an ideal anatomical condition) back into the syringe [76].

### 4.3. Preparation and Storage of Samples

Plasma, PF, and PW were centrifuged at 2000× *g* at 4 °C for 15 min to remove any residual cellular contamination. Part of the supernatant (700 µL) was analyzed during the same day by LS. Supernatants of ascites, PF and PW were always analyzed before and directly after being slowly and carefully filtered through 0.22 µm filters (Millex-GP, Millipore, Ireland) directly into glass cuvettes for LS measurements to remove eventual larger particles, which were sometimes floating in the samples and could influence/disturb the measurement.

To evaluate the effect of time from sampling to analysis of results, ascites and plasma were analyzed immediately and 1 and 3 days after the sampling. The PF and PW were analyzed immediately and on the first day only; longer times were not considered in this case because of the instability of the samples. The remaining amount of the supernatants obtained by centrifugation was separated into smaller aliquots, put to −20 °C for 24 h and then stored at −80 °C. To analyze the effect of freezing, fresh and thawed samples of plasma, ascites, PF, and PW were also analyzed by LS. Samples were thawed at 4 °C.

Due to the lower LS intensity of PF, and particularly of PW, we tried to concentrate the NPS in these types of samples using differential ultracentrifugation according to the protocols for exosome enrichment [58,77]. PF (13 mL) and PW (13 mL) of thawed samples from patient BP-E were ultracentrifuged and analyzed during the same day (see Supplementary Materials for LS results on ultracentrifuged samples).

#### Isolation/Concentration of Exosomes by Differential Ultracentrifugation

Thawed supernatant obtained after centrifugation at low speed was centrifuged at 12,000× *g* at 4 °C for 45 min and then pelleted by ultracentrifugation at 110,000× *g* at 4 °C for 120 min. The pellet was re-suspended in 1 mL of phosphate buffer solution (PBS) and then diluted with a large volume of PBS. The resuspension was passed through a 0.22 µm filter and ultracentrifuged again at 110,000× *g* at 4 °C for 70 min. The obtained pellet was re-suspended in 700 µL of PBS for subsequent LS analysis.

### 4.4. Static and Dynamic Light Scattering Measurements

DLS and SLS measurements were used to determine the hydrodynamic radius (*R*_h_) and the radius of gyration (*R*_g_) of particles in samples. All LS measurements were conducted with the 3D-DLS-SLS cross-correlation spectrometer from LS Instruments GmbH (Fribourg, Switzerland) using a 20 mV He-Ne laser (Uniphase JDL 1145P) with a wavelength λ_o_ = 632.8 nm as the light source. All details regarding the instrumentation were reported previously [26,37,42,43].

Correlation functions and the integral time averaged intensities were recorded simultaneously. The intensities were normalized with respect to the Rayleigh ratio of toluene and converted into the absolute intensity units (cm^−1^). Together with the absolute LS intensity of the samples (*R*), the absolute LS intensity of the solvent (*R*_0_) was measured, which enabled the calculation of the excess LS intensity of the samples, expressed as ∆*R* (=*R* − *R*_0_). To calculate Δ*R*, water was chosen as the solvent. All measurements were performed in the angular range from 30° to 150° with a step of 20° after equilibrating the samples at 25 °C for 15 min. Constant intensity of light scattered at 90° was used as the criterion that the solution was properly equilibrated. Five intensity correlation functions were collected at each angle and averaged. Each curve was analyzed independently and compared with the averaged curve to ensure accuracy of the mathematical solution.

Detailed methodological aspects of SLS and DLS can be found elsewhere [38,39]. Additional experimental procedures regarding the LS measurements together with the procedures used for data treatment in this paper are described in detail in SI. A brief explanation is given below.

In DLS, the fluctuations of the intensity of scattered light are monitored in dependence on time. This information is presented in the form of a correlation function of the scattered light intensity, $G_{2}\left(t\right)=\langle I\left(0\right)I\left(t\right)\rangle$, where *t* is the time on the relaxation time axis and *I*(0) and *I*(t) are the intensities of scattered light at *t* = 0 and at an arbitrary time *t*, respectively. To determine the diffusion coefficient (*D*) of a Brownian particle, the *G*_2_(*t*) function is converted into the correlation function of the scattered electric field (*g*_1_(*t*)) using the Siegert’s relationship. The function *g*_1_(*t*) is related to *D* by

(1) $$\left|g_{1}\left(t\right)\right|=e^{-t/\tau }=e^{-\Gamma t}=e^{-Dq^{2}t}$$

where *q* (=$(4{\pi n}_{0}/\lambda_{0})\sin\left(\theta /2\right)$) is the scattering vector that depends on the wave length of the incident light (*λ*_0_), the scattering angle (θ), and the refractive index of the medium (*n*_0_; *n*_0_ = 1.333 for water at 25 °C), *τ* is the relaxation (or decay) time, and Γ (= *τ*^−1^ = *Dq*^2^) is the relaxation (or decay) rate. The value of *R*_h_ is then obtained from *D* via the Stokes-Einstein equation:

(2) $$R_{h}=\frac{kT}{6\pi \eta_{0}D}$$

where *k* is the Boltzmann constant, *T* is the absolute temperature, and *η*_o_ is the viscosity of the solvent. The viscosity of the medium, *η*_0_, is a critical parameter in the correct *R*_h_ determination of particles in plasma. In most of the previous studies on exosomes and other particles (vesicles) in plasma, the viscosity of distilled water (*η*_o_ = 0.90 mPa s^−1^ at 25 °C) was used for *R*_h_ calculation from Equation (2). This is clearly an oversimplification (large approximation).

In a recent study, Božič et al. [26] estimated the *η*_o_ value of the medium of blood plasma by direct viscosity measurements and arrived at a value *η*_o_ ≈ 1.2 mPa s^−1^ (at 25 °C), which is around 32% higher than that of pure water. It was shown there [26] that this *η*_o_ is appropriate for the calculation of *R*_h_ when particles’ sizes exceeded ~100 nm; whereas, for smaller particles (sizes below ~50 nm), the viscosity of water was shown to be suitable. The same approach is used in our study for determination of *R*_h_ of large particles in OC patients. On the other hand, this correction was not considered for larger particles in body fluids of BP patients because those fluids were much less dense and less viscous. Their viscosity was close to water’s.

Equation (1) (the mono-exponential form) is strictly valid for monodisperse spherical particles. For polydisperse particles, several exponents appear in this expression, and a multi-exponential fit of *g*_1_(*t*) has to be performed. The multi-exponential fit was achieved by the inverse Laplace transformation program CONTIN, resulting in the distribution of the relaxation times of species in solution. The distributions over *τ* were converted into *R*_h_ distributions by means of Equation (2).

For the herein studied samples, all size distributions were multimodal, exhibiting two to four peaks. An example of such an *R*_h_ distribution is reported in Figure S1 (Supplementary Materials) together with the method of calculating the contributions of the peaks referring to exosomes (i.e., peaks 2 and 3) to the total LS intensity. The time-averaged intensities associated with these two peaks were then treated separately for the angular dependency to determine the *R*_g_ of the scattering particles. The latter was obtained from the form factor (*P*(*q*)) defined as:

(3) $$P\left(q\right)=\frac{I\left(q\right)}{I\left(0\right)}=\frac{1}{1+\frac{{\left(qR_{g}\right)}^{2}}{3}}=1-\frac{{\left(qR_{g}\right)}^{2}}{3}$$

where *I*(0) is the scattering intensity at θ (or *q*) = 0. Details of this procedure can be found in Supplementary Materials and in the literature [40,41,42,43]. The expression for *P*(*q*) as given by the right-hand side of Equation (3) is known as the Zimm function [39,64] and is valid for particles that are small in comparison with the wavelength of light (fulfilling the criterion *qR*_g_ < 1.5), which was the case for almost all particles treated in this study. Either first (for *R*_g_ ≈ 25 nm; peak 2; Equation (3)) or second (for higher *R*_g_ values, peak 3) order fit was used to fit the *P*(*q*) as a function of *q*^2^ (*c.f.* the right-hand side of Equation (S7a)).

The particle size characteristics obtained by SLS and DLS, i.e., *R*_g_ and the zero angle *R*_h_ values (*R*_h,0_), respectively, were combined to give so-called shape parameter ρ (=*R*_g_/*R*_h,0_). The ρ-ratio provides an important indication of the scattering particle topology (shape), especially for the relatively small particles as studied herein. Some theoretically calculated values of ρ for the most important particle shapes are reported in Table S2 (Supplementary Materials). For the present study, the following ρ-values are noteworthy: ρ of a hard (homogeneous) sphere is 0.778 and that of a hollow sphere, which is the most appropriate shape approximation for various vesicles, is 1.

## 5. Conclusions

The structural data collected in this paper for the ENPs from body fluids of patients diagnosed with advances serous ovarian cancer (OC) are the first of their kind. Using SLS and DLS, we characterized and compared the sizes and structural characteristics of ENPs derived from OC patients with those from the control group, which enrolled patients with common benign gynecological pathology (BP).

The majority ENP population in the plasma and ascites of OC patients demonstrated a hydrodynamic radii of around 25 nm (therefore, termed smaller ENPs), and the value of the shape parameter ρ for these ENPs was, on average, around 1 (ρ = 0.97–1.16). Larger ENPs (*R*_h,0_ close or above 100 nm) were found mainly in samples that underwent freezing and subsequent thawing and displayed similar ρ values (ρ = 0.99–1.15) to the small ones. Their larger size may be due to membrane rapture upon freezing and the repeated organization of lipids into curved bilayer structures upon thawing. It is, therefore, recommended that measurements be performed on fresh samples, which is not always possible.

The ρ = 1 value is characteristic for monodisperse hollow spheres, which is the proposed shape of vesicles. The polydispersity of both ENP populations contributes to positive deviations of the calculated form factor from the theoretical curve for a hollow sphere. This, together with the relatively small size and expected heterogeneity of ENPs in most OC samples, is responsible for the fact that the static light scattering data do not unambiguously follow the form factor curve of vesicles.

Although EVs, which are probably among the more numerous particles in this ENP population, are supposed to contain some internal cargo, the OC-derived ones appeared to have their mass predominately concentrated on the rim with a less dense interior (resembling a dilute solution of ions, sugars, small proteins, and alike). We presume that their small size (*R*_h_ ≈ 20–30 nm) does not allow a more extensive incorporation of larger particles into their interior.

The DLS and SLS results for the BP patients did not allow such straightforward conclusions. In comparison with OC patients, the data for BP patients were much more scattered, and differentiation between two ENPs populations was not always possible. The average size (*R*_h_) of ENPs in plasma of BP patients is in the same range as for the OC patients, and their contribution to the total LS intensity was also high; however, the analysis of *R*_g_ was mostly not possible and, therefore, the shape parameter was difficult to evaluate.

On the other hand, sizes of ‘peak 2′ ENPs found in PF and PW of BP patients were, on average, larger than those in local fluids of OC patients, and so was the value of ρ (from ~1 to ~2). These ρ values suggest a deformation of the EV shape from spherical to more elongate, which seems a realistic scenario for larger ENPs in dilute suspensions, such as PF and PW.

It needs to be stressed that the obtained structural characteristics are population averaged and that individual particles in the complex samples can be (and were) morphologically heterogeneous. However, the overall ENPs scattering profile seems to be carrying information about the state of the organism that might be useful in the OC diagnostics. We conclude that SLS and DLS are techniques that present significant advantages for the characterization of size and shape (topology) of ENPs in plasma and ascites of OC patients and may have the potential to discriminate between OC and BP patients.

## Figures and Tables

**Table 1:** The mean *R*_h_ values for the peak positions in size distributions of plasma, ascites, and filtered ascites (ascitesF) of ovarian cancer patient OC1 determined at θ = 90° and *T* = 25 °C.

	*R*_h_/nm	Origin
Peak 1	<10	various proteins
Peak 2	15–35	smaller ENPs
Peak 3	>50	larger ENPs
Peak 4	several 100–1000	larger aggregates

**Table 2:** Structural data for ENPs (represented by peak 2 and 3) determined by dynamic (DLS) and static light scattering (SLS) measurements for patients with ovarian cancer (OC): the mean hydrodynamic radius for peaks in the size distributions at an angle θ = 90° (*R*_h,90_), hydrodynamic radius extrapolated to θ = 0° (*R*_h,0_), radius of gyration (*R*_g_), the shape parameter $\rho (=R_{g}/R_{h})$, and the contribution of by peak 2 (or 3) particles to the total scattering intensity at θ = 90° (% *I*_tot,90_). The *R*_h_ values in bold and italic were obtained with *η*_o_ ≈ 1.2 mPa s^−1^.

		*R*_h,90_/nm			Angular Dependency (Peak 2 or 3)			Peak 2 or 3
Patient	Sample	Peak 1	Peak 2	Peak 3	*R*_h,0_/nm	*R*_g_/nm	ρ	% *I*_tot,90_ (Peak 2 or 3)
**OC1** ^#^	plasma	3.7	24	70				
	ascites	7.0	27	80				
	ascitesF	/	29	/				
**OC2**	plasma	5.4	24	/	24.3	24.8	1.02	88
	ascites	4.2	21	/	22.9	23.7	1.04	77
	ascitesF	4.0	20	/	20.2	20.4	1.01	76
**OC3**	plasma	4.4	17	46 ^&^	25.4	26.2	1.03	63
	ascites	7.2	17	/	20.6	21.1	1.03	61
	ascitesF	4.6	20	/	21.9	25.5	1.16	77
**OC4**	plasma	2.7	22	/	24.7	25.5	1.03	64
	ascites	2.9	17	102 ^&^	21.9	21.2	0.97	51
	ascitesF	4.1	24	/	21.2	21.8	1.03	56
**OC5**	plasma	4.7	23	/	34.0	34.0	1.00	50
	ascites	3.7	20	/	34.2	36.3	1.06	46
	ascitesF	4.1	24	/	46.8	53.4	1.14	43
**OC6**	plasma	5.1	26	/	24.6	24.6	1.00	74
	ascites	4.7	21	/	28.0	29.2	1.04	49
	ascitesF	4.0	18		22.9	22.7	0.99	63
				***68***	***150***	***160***	***1.07***	***13***
	ascites *	5.6	27		28.7	29.0	1.01	18
				***150***	***143***	***165***	***1.10***	***15***
	ascitesF *	3.8	14		17.0	36.0	(~2.1)	56
				***62***	***101***	***115***	***1.14***	***34***
**OC7**	plasma *	4.1	20		~21	~32	(~1.5)	76
				***173***	***188***	***185***	***0.99***	***11***
	ascites *	3.2	16		26.9	28.4	1.05	71
				***83***	***203***	***230***	***1.13***	***13***
	ascitesF *	4.5	20		22.3	33.4	(~1.5)	75
				***90***	***105***	***115***	***1.09***	***13***

^#^ for patient OC1 only preliminary DLS measurements at θ = 90° were collected; * analysis of thawed samples; ascitesF = filtered ascites; ^&^ for these particles, the analysis of angular dependency could not be performed; values in bold and italic in the “angular dependency” column are for peak 3.

**Table 3:** Structural data for ENPs (represented by peak 2) determined by dynamic (DLS) and static light scattering (SLS) measurements for patients with benign ovarian cysts (BP-BOC): the mean hydrodynamic radius for peaks in the size distributions at an angle θ = 90° (*R*_h,90_), the hydrodynamic radius extrapolated to θ = 0° (*R*_h,0_), the radius of gyration (*R*_g_), the shape parameter $\rho (=R_{g}/R_{h})$, and the contribution of peak 2 particles to the total scattering intensity at θ = 90° (*I*_tot,90_).

		*R*_h,90_/nm			Angular Dependency (Peak 2)			
Patient	Sample	Peak 1	Peak 2	Peak 3	*R*_h,0_/nm	*R*_g_/nm	ρ	% *I*_tot,90_
**BP-BOC1**	plasma	6.1	31	/	~29	/ ^#^	/ ^#^	89
	PF	4.7	19 ^&^	123	/ ^&^	/ ^#^	/ ^#^	25
**BP-BOC2**	plasma	3.3	17	107	24	26	1.1	76
	PF	2.9, 7.7	31	/	51	67	1.3	18
**BP-BOC3**	plasma	4.3	18	102	~20	/ ^#^	/ ^#^	56
	PF	3.7, 13.5	42	390	36	68	1.9	24
**BP-BOC4**	plasma	2.8, 7.2	36	/	28	/ ^#^	/ ^#^	69
	PF	3.3	13	63.0	29	55	1.9	23
**BP-BOC5**	plasma	3.7	24	/	23	28	1.2	63
	PF	3.1, 10	34	/	40	51	1.3	22

^#^ analysis of the angular dependency was not possible; ^&^ this peak likely does not correspond to ENPs; PF = peritoneal fluid.

## Data Availability

All the data is included within the article and as Supporting Material.

## Supplementary Materials

The following are available online at https://www.mdpi.com/article/10.3390/ijms222312946/s1.
