# Supplementary material for: Application of Dynamic and Static Light Scattering for Size and Shape Characterization of Small Extracellular Nanoparticles in Plasma and Ascites of Ovarian Cancer Patients
Source: Int J Mol Sci. 2021 Nov 30;22(23):12946. doi: 10.3390/ijms222312946 (PMC8657631; doi:10.3390/ijms222312946)
Supplement: Supplementary file 1 [file ijms-22-12946-s001.zip › ijms-1493300-supplementary.pdf]

Supplementary Materials for the paper:

Application of Dynamic and Static Light Scattering for Size and Shape Characterization of Extracellular Nanoparticles in Plasma and Ascites of Ovarian Cancer Patients

Ksenija Kogej [1,*], Darja Božič [1,2], Borut Kobal [3,4], Maruša Herzog [3,4] and Katarina Černe [5]

[1] University of Ljubljana, Faculty of Chemistry and Chemical Technology, Department of Chemistry and Biochemistry, Ljubljana, Slovenia; ksenija.kogel@fkkt.uni-lj.si
[2] University of Ljubljana, Faculty of Health Sciences, Laboratory of Clinical Biophysics, Ljubljana, Slovenia; darja.bozic@zf.uni-lj.si
[3] University Medical Centre Ljubljana, Department of Gynecology, Division of Gynecology and Obstetrics, Ljubljana, Slovenia; borut.kobal@kclj.si, marusa.herzog@kclj.si
[4] University of Ljubljana, Faculty of Medicine, Department of Gynecology and Obstetrics, Ljubljana, Slovenia;
[5] University of Ljubljana, Faculty of Medicine, Department of Pharmacology and Experimental Toxicology, Ljubljana, Slovenia; katarina.cerne@mf.uni-lj.si

* Correspondence: Ksenija Kogej, ksenija.kogel@fkkt.uni-lj.si

Material and Methods

Patients

The study included 7 patients with advanced OC [FIGO IIIC-IV] and 7 patients with BP of reproductive organs as a control group (see the list of patients in Table S1), who were operated between February 2015 and January 2016 at the Department of Gynecology, University Medical Centre of Ljubljana. Out of seven BP patients, the control group enrolled five patients with benign ovarian cyst (designation BP-BOC), one with myoma of the uterus (patient BP-M) and one with endometriosis (patient BP-E). The clinical characteristics of patients are reported in Table S1. Using FIGO criteria for surgical staging, all OC patients were diagnosed with advanced disease (stage IIIC-IV). All patients had the same histopathological subtype of OC (serous carcinoma) and poorly differentiated tumor tissue (histological grade G3). CA-125, standard tumor marker for OC, was elevated in all patients. According to markers of inflammation, six patients had elevated CRP and three patients had elevated WBC count. CA-125 was elevated in the BP-E patient and CRP was elevated in one patient with BOC (BP-BOC3). In OC patients, plasma and ascites were collected, whereas in BP patients, peritoneal fluid (PF) and peritoneal washing (PW), which can replace PF as a control of the local environment in such situations, were collected [1-3]. PW has already been incorporated in the International Federation of Gynaecology and Obstetrics (FIGO) staging classification for OC.

Table S1. Designation of patients and their clinical characteristics.

Patient	Diagnosis	Histopathology	CA-125 [U/ml]	FIGO stage	Histol. grade	CRP [mg/L]	WBC [x10⁶/L]	Ascites/*PT* [mL]
OC1	ovarian cancer	serous carcinoma	9851	IV	G3	62	13.4	6400
OC2	ovarian cancer	serous carcinoma	2415	IIIC	G3	211	11.9	2000
OC3	ovarian cancer	serous carcinoma	732	IIIC	G3	60	8,8	9000
OC4	ovarian cancer	serous carcinoma	4997	IIIC	G3	101	12.7	120
OC5	ovarian cancer	serous carcinoma	263	IIIC	G3	7	5.9	600
OC6	ovarian cancer	serous carcinoma	155	IIIC	G3	<5	6.1	150
OC7	ovarian cancer	serous carcinoma	642	IIIC	G3	130	9.1	7000
BP-BOC1	benign ovarian cyst	adenofibroma	11	NA	NA	<5	4.9	*5*
BP-BOC2	benign ovarian cyst	serous cystadenoma	13	NA	NA	<5	4.4	*26*
BP-BOC3	benign ovarian cyst	serous cystadenoma	15	NA	NA	34	7.9	*0,7*
BP-BOC4	benign ovarian cyst	endometrioma	21	NA	NA	<5	6.4	*18*
BP-BOC5	benign ovarian cyst	teratoma	15	NA	NA	<5	3.8	*15*
BP-M	myoma of the uterus	leiomyoma	14	NA	NA	<5	6.3	*10*
BP-E	endometriosis	peritoneal endometriosis	71	NA	NA	<5	5.6	*43*

OC = ovarian cancer; BOC = benign ovarian cyst; BP = benign pathology M = myoma of the uterus; E = endometriosis; CA = carbohydrate antigen (standard ovarian tumor marker, cut off value ≥ 35 U/mL); FIGO = International Federation of Gynecology and Obstetrics; CRP = C-reactive protein (normal level: <5); WBC = total white blood cells count (normal range: 4.0-10 x 10^{-6} L); *PT* = peritoneal fluid; NA = not applicable.

Light Scattering

Dynamic, DLS, and dynamic static light scattering, SLS, experiments were used to determine the hydrodynamic radius, R_h, and the radius of gyration, R_g, of particles in samples. All LS measurements were conducted with the 3D-DLS-SLS cross-correlation spectrometer from LS Instruments GmbH (Fribourg, Switzerland) [4]. As a light source, the 20 mV He-Ne laser (Uniphase JDL 1145P) with a wavelength λ_o = 632.8 nm was used. Two coherent incident light beams were generated, and the scattered light was collected by two detectors, enabling the correlation of the two scattered beams with each other. Measurements were performed in the angular range from 30° to 150° with a step of 20° after equilibrating the samples at 25 °C for 15 minutes. Constant intensity of light scattered at 90° was used as the criterion that the solution was properly equilibrated. Five intensity correlation functions were collected at each angle and averaged. Each curve was analyzed independently and compared with the averaged curve to ensure accuracy of the mathematical solution. Detailed methodological aspects of SLS and DLS can be found elsewhere [5,6]. In the following, we describe the procedures used for data treatment in our approach.

In DLS, fluctuations of the intensity of scattered light are monitored in dependence on time. Intensity fluctuations originate from the concentration fluctuations arising from the Brownian motion of particles in solution. The result of the DLS experiment is the correlation function of the scattered light intensity, $G_2(t)$ (t is the time on the relaxation time axis), defined as:

$$G_2(t) = \langle I(0)I(t)\rangle \tag{S1}$$

where $I(0)$ and $I(t)$ are the scattering intensities at time t = 0 and time t, respectively. Similarly, the correlation function of the scattered electric field, $g_1(t)$, is defined as:

$$g_1(t) = \langle E(0)E(t)\rangle \tag{S2}$$

where $E(0)$ and $E(t)$ are the corresponding scattering amplitudes at t = 0 and t, respectively. The $g_1(t)$ function is directly related to the diffusion coefficient, D, of a Brownian particle by:

$$|g_1(t)| = e^{-t/\tau} = e^{-\Gamma t} = e^{-Dq^2 t} \tag{S3}$$

In Eq. S3, q (= $(4\pi\, n_0/\lambda_0) sin(\theta/2)$) is the scattering vector, depending on the wave length of the incident light (λ_o), the scattering angle (θ), and the refractive index of the medium (n_o), τ is the relaxation (or decay) time and Γ (= $\tau^{-1} = Dq^2$) is the relaxation (decay) rate. Since the intensity and the amplitude of the electric field are related through $I = |E|^2$, the relation between the correlation functions $G_2(t)$ and $g_1(t)$ is usually written as:

$$G_2(t) = 1 + Bg_1^2(t) \tag{S4a}$$

where B is an experimental constant. Eq. S4a enables the transformation of $G_2(t)$ into $g_1(t)$, from which D is calculated by means of Eq. S3. The value of R_h is then obtained from D via the Stokes-Einstein equation:

$$R_h = \frac{kT}{6\pi\eta_0 D} \qquad \text{(S5)}$$

Here, k is the Boltzmann constant, T the absolute temperature, and η_o the viscosity of the solvent. For n_o and η_o values for water at 25 °C were used. Note that in Figures S2 and S3 and in the manuscript, the correlation function $G_2(t)$ is plotted in the normalized form:

$$|G_2(t)| = \frac{G_2(t) - G_2(\infty)}{G_2(0) - G_2(\infty)} = |G_2(t)|_0^1 \qquad \text{(S4b)}$$

For simplicity, it is written as $G_2(t)$.

For polydisperse particles, several exponents appear in Eq. S3 and thus a multi exponential fit is used for $g_1(t)$ and the corresponding values of τ (or D) are determined by the inverse Laplace transform program CONTIN developed by Provencher [7]. The result of such procedure is the distribution of the relaxation time of species in solution. The distributions over τ are then converted into size distributions (over R_h) by means of Eq. S5. For the herein studied samples all size distributions were multimodal, exhibiting 2-4 peaks. The mean R_h values for the peak positions determined at $\theta = 90°$ and their origin are reported in Table 2 in the main paper.

Simultaneously with the correlation functions, the integral time averaged intensities, $I(\theta) \equiv I(q)$, were recorded in the θ-range from 30 to 150°. Intensities measured in counts of photons per second (cps) were normalized with respect to the Rayleigh ratio, R, of toluene in and converted into the absolute intensity units given in cm^{-1}. Measurements of the angular dependency of scattered light enable the determination of the form factor, $P(q)$, of colloidal particles in solution. $P(q)$ is defined as:

$$P(q) = \frac{I(q)}{I(0)} \qquad \text{(S6)}$$

where $I(0)$is the scattering intensity at θ (or q) = 0. From $P(q)$, the radius of gyration, R_g, of particles, was obtained.

Our aim was to determine the structural characteristics (R_h and R_g) of exosomes (R_h around 25 nm), which were normally peak 2 in the size distributions (c.f. Table 2 in the main paper). For this purpose, the calculated distributions of the relaxation times were used to extract the contribution of peak 2 to the total intensity of scattered light. These data were then treated separately for the angular dependency. Details of this procedure can be found elsewhere [8]. In some cases, the contribution of peak 3 ($R_h > 100$ nm; see Results) to the total LS intensity could be extracted as well and these data were also analyzed for angular dependency.

In view of the small size of exosomes ('peak 2' particles; see Results in the main paper, Tables 2 and 3), the Zimm function

$$P(q) = \frac{1}{1 + \frac{(qR_g)^2}{3}} = 1 - \frac{(qR_g)^2}{3} \qquad \text{(S7)}$$

was used to determine the R_g values. This function is valid for particles that are small in comparison with the wavelength of light, *i.e.*, that fulfill the criterion $qR_g < 1.5$, which was the case with almost all

particles. Either first (for $R_g \approx 25$ nm; peak 2) or second (for higher R_g, peak 3) order fit was used to fit the $P(q)$ as a function of q^2 according to Eq. S7a:

$$P(q) = 1 - \frac{(qR_g)^2}{3} + \left(\frac{(qR_g)^2}{3}\right)^2 \qquad \text{(S7a)}$$

A useful scattering function for globular structures having a spherical distribution of points around the center of gravity with the Gaussian probability is also the Guinier function:

$$P(q) = e^{-\frac{(qR_g)^2}{3}} \qquad \text{(S7b)}$$

The form factor of a hollow sphere with radius R and with a very thin shell, which is the most suitable shape approximation for vesicles, has the form

$$P(q) = \left[\frac{sin(qR)}{qR}\right]^2 \qquad \text{(S7c)}$$

and finally, the form factor for a homogeneous solid spherical particle with radius R is

$$P(q) = \left[\frac{3}{(qR)^3}\big(sin(qR) - (qR)cos(qR)\big)\right]^2 \qquad \text{(S7d)}$$

Form factors $P(q)$ given by equations S7 are presented in Figure 4 in the main manuscript as solid lines. Other details on these functions can be found also in SM of the paper Božič et al. [9].

The particle size characteristics determined by SLS and DLS, *i.e.*, R_g and $R_{h,0}$ (at $\theta = 0°$), respectively, can be combined to give so-called shape parameter, ρ:

$$\rho = \frac{R_g}{R_{h,0}} \qquad \text{(S8)}$$

The ρ-ratio provides an important indication of the scattering particle morphology (shape) [5], especially for the relatively small particles as studied in here. Theoretically calculated values of this parameter for some selected particle shapes that are relevant for the herein studied particles are reported in Table S2. For the present study the following ρ-values are noteworthy: ρ of a monodisperse homogeneous hard sphere is 0.778 and the of a monodisperse hollow sphere with a higher mass density on the surface and a lower one in the center (*e.g.*, vesicles, exosomes) is 1.

Table S2. The shape parameter, ρ, for some most typical particle topologies/shapes [5].

Topology	ρ
rigid rod	>2
random polymer coil#	1.5 – 1.8 (2)
hollow sphere*	1.0
rigid homogeneous sphere	0.78
microgels§	~0.6

#a more precise value of ρ depends on solvent quality, structural characteristics of the polymer chain and temperature.
*particles with a higher mass density on the surface and a lower one in the center (e.g. vesicles, exosomes).
§particles with a higher mass density in the center and a lower one towards the surface.

To calculate the radii of gyration of the scattering particles represented by peaks 2 and 3 (designations R_h^{peak2} and R_h^{peak3}, respectively; *c.f.* Figure S1), the total LS intensity was split into two contributions, $I_2(q)$ and $I_3(q)$. The splitting is based on the intensity weighted distributions of the relaxation times τ. Each scattering species of the radius $R_{h,i}$ is represented in this distribution by its relative contribution, *i.e.* by the amplitude $A_i(\tau_i)$, to the total intensity of scattered light $I_t(q)$. The relative amplitudes of the two peaks are then estimated using the procedure outlined in reference 5 as

$$A_2 \equiv \sum_{peak2} A_i(\tau_i) \qquad \text{and} \qquad A_3 \equiv \sum_{peak3} A_i(\tau_i) \tag{S9}$$

and converted into the LS intensities for each population of particles. The time-average intensities of peaks 2 and 3, $I_2(q)$ and $I_3(q)$, respectively, are calculated as:

$$I_2(q) = A_2(q) \cdot I_t(q) \qquad \text{and} \qquad I_3(q) = A_3(q) \cdot I_t(q) \tag{S10}$$

An example of such multimodal distribution is shown in the Figure S1, where the $A_i(\tau_i)$, values at a particular $R_{h,i}$ are indicated. The intensities of the peaks obtained by the outlined procedure were then analyzed separately for the angular dependency.

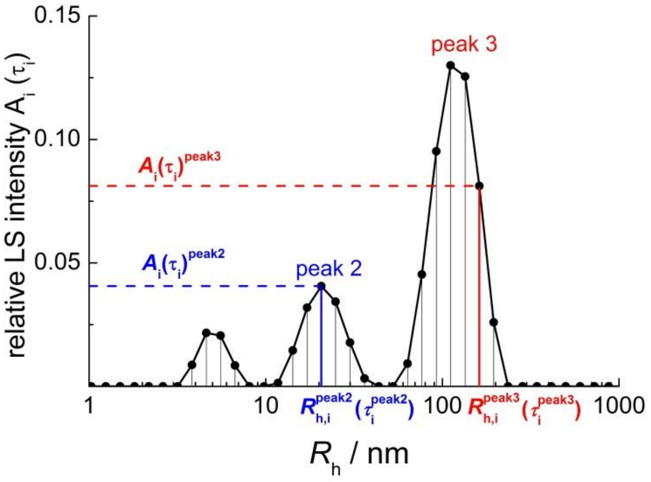

Figure S1. Intensity weighted distribution of the hydrodynamic radii, R_h, measured for plasma of ovarian cancer patient OC5 at θ = 90° and T = 25°C. The Y-axis shows the relative contributions of particles of size $R_{h,i}$, $A_i(\tau_i)$, to the total LS intensity.

RESULTS

1. *Preliminary DLS measurements at* $\theta = 90°$

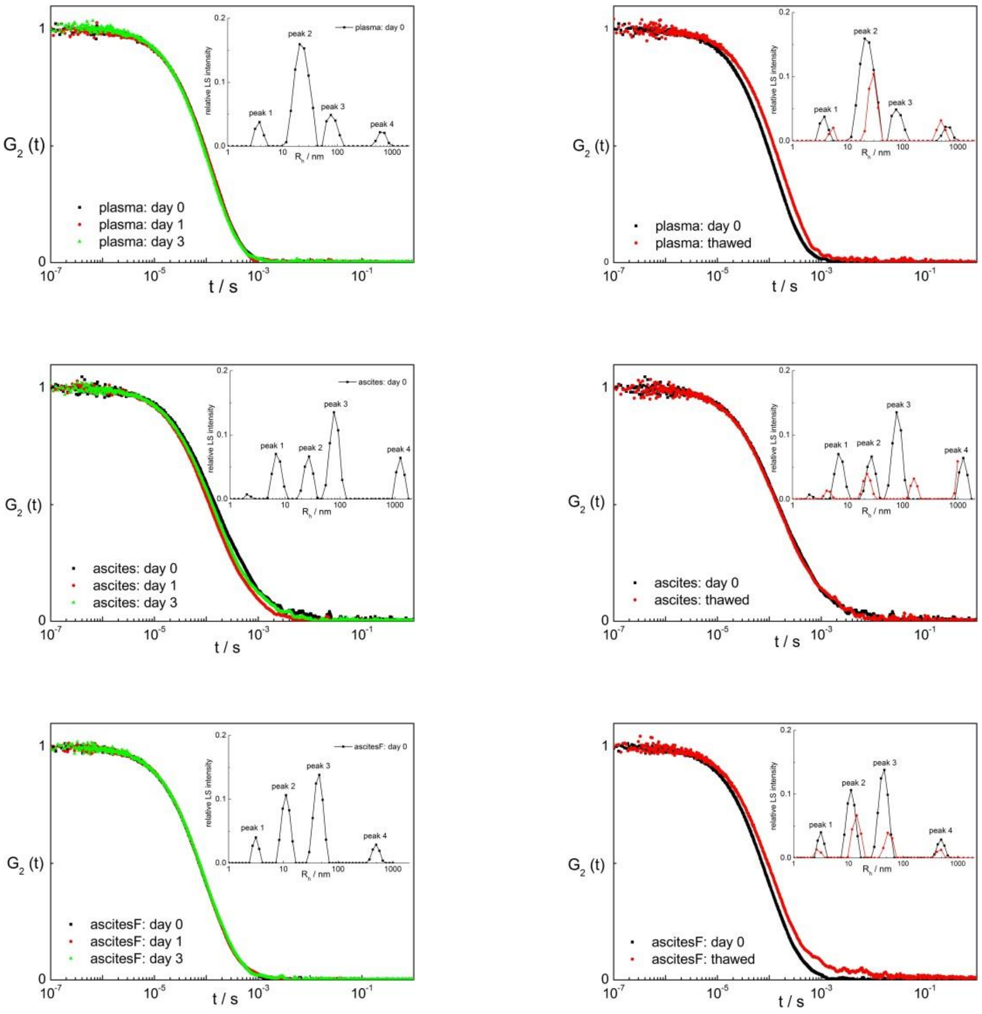

Figure S2. *Left panel*: the normalized intensity correlation functions (G_2(t); see Eq. S4b) of plasma, unfiltered ascites, and filtered (ascitesF) for ovarian cancer (OC) patient OC1 at various times: on the day of sampling (day 0) and after 1 (day 1) and 3 days (day 3). In the insets, the calculated size distributions on day 0 are shown. *Right panel*: correlation functions G_2(t) of samples on day 0 and after freezing and thawing. In the insets, the calculated size distributions (day 0) are shown. All measurements were performed at θ = 90° and 25°C.

Table S3. Mean R_h values for peak positions in size distributions of plasma and ascites for ovarian cancer (OC) patient OC1 determined by DLS at θ = 90° and 25°C.

sample		$\bar{R}_h$ / nm		
		peak 1	peak 2	peak 3
plasma	day 0	3.7	24	71
	day 1	4.5	29	
	day 3	6.6	34	
	thawed	*6.1*	*29*	
ascites	day 0	2.1, 7.3	27	80
	day 1	3.6	12	63
	day 3	3.3	17	83
	thawed	*4.4*	23	*160*
acitesF	day 0	3.1	11	44
	day 1	3.5	17	
	day 3	5.0	29	
	thawed	*2.8*	*14*	*55*

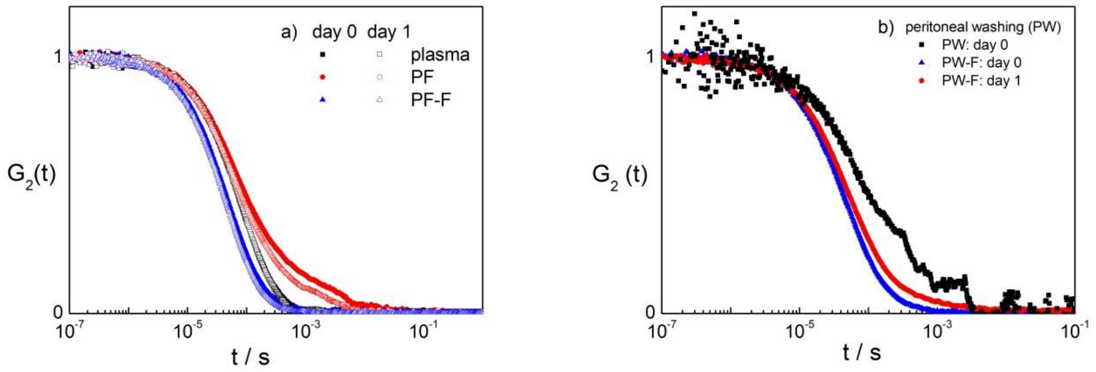

Figure S3. The normalized intensity correlation functions of a) plasma and peritoneal fluid (PF) and b) peritoneal washing (PW) of patient BP-M with myoma of the uterus (M) as benign pathology (BP) of reproductive organs on day 0 and 1; at θ = 90° and *T* = 25°C; F = filtered.

Table S4. Mean R_h values for the peak positions in size distributions of plasma, peritoneal fluid (PF), and peritoneal washing (PW) for patient BP-M with myoma of the uterus (M) as benign pathology (BP) of reproductive organs as determined by DLS at θ = 90° and 25°C.

sample		$\bar{R}_h$ / nm		
		peak 1	peak 2	peak 3
plasma	day 0	6	23	
	day 1	3, 10	28	110
PW	day 0	9	/	240
PW-F	day 0	12	/	100
	day 1	11	/	157
PF	day 0	6	25	110
	day 1	5	19	310
PF-F	day 0	3, 9	24	
	day 1	3, 11	67	

F = filtered

2. *Angular dependency and the determination of R_g*

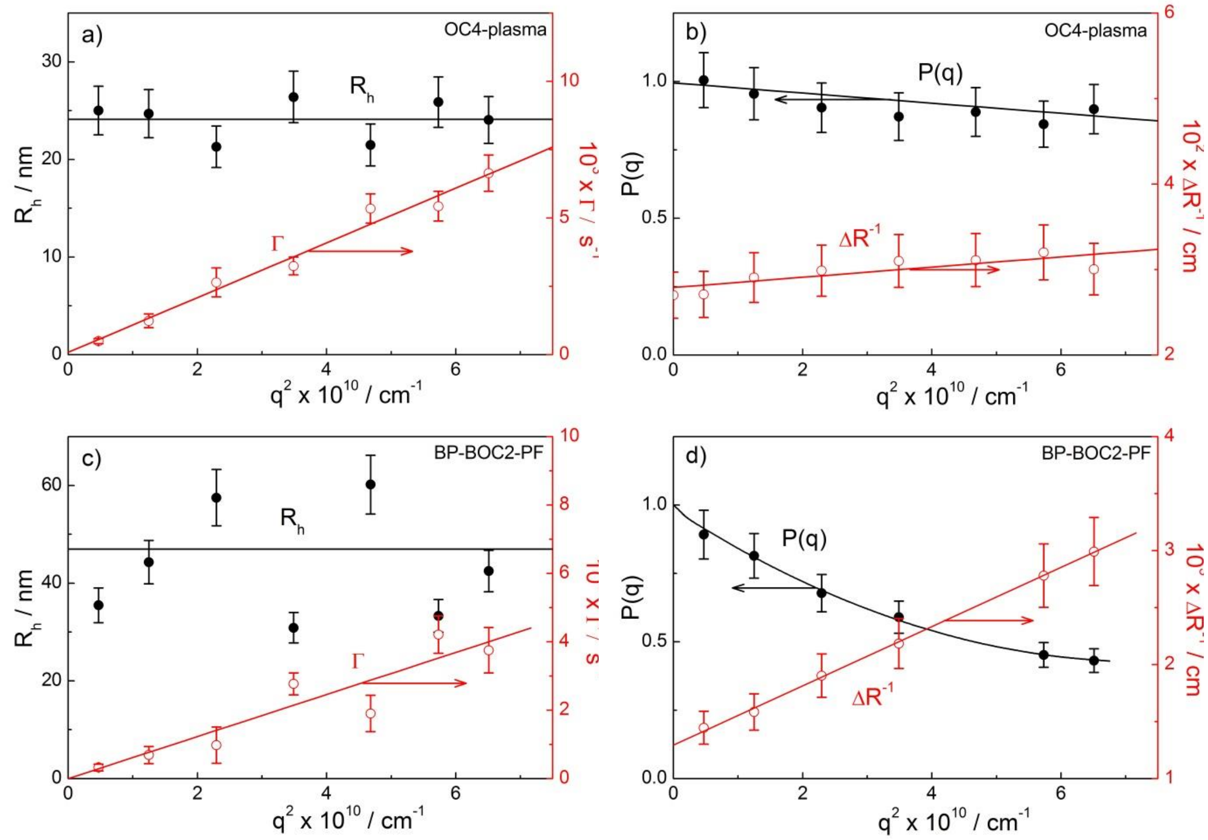

Figure S4. a) and c) Hydrodynamic radii, R_h (filled circles), and decay rates, Γ (empty red circles) and b) and d) form factor, *P*(q), and the excess Rayleigh ratio, Δ*R*, as a function of the square of the scattering vector, q^2, for EVs in plasma of ovarian cancer patient OC4 (a and b) and in peritoneal fluid (PF) of patient BP-BOC2 with benign ovarian cyst (BOC) as benign pathology (BP) of reproductive organs (c and d).

Table S5. Structural data for large ENP population determined by dynamic (DLS) and static light scattering (SLS) measurements for patients OC6 and OC7: the mean hydrodynamic radius for peaks in the size distributions at an angle θ = 90° ($R_{h,90}$), hydrodynamic radius extrapolated to θ = 0° ($R_{h,0}$), radius of gyration (R_g), the shape parameter $\rho (= R_g/R_h)$, and the contribution of EVs to the total scattering intensity at θ = 90° (% $I_{tot,90}$). The grey highlighted R_h and ρ values given in bold and italic outside parenthesis are obtained with viscosity of water (η_o = 0.9 mPa s^{-1}), and those inside parenthesis with viscosity of the plasma medium (η_o = 1.2 mPa s^{-1}; see ref Božič et al. [9] and main paper).

		$R_{h,90}$ / nm			angular dependency (peak 2 or 3)			
patient	sample	peak 1	peak 2	peak 3	$R_{h,0}$ / nm	R_g / nm	ρ	% $I_{tot,90}$
OC6	plasma	5.1	26	/	24.6	24.6	1.00	74
	ascites	4.7	21	/	28.0	29.2	1.04	49
	ascitesF	4.0	18		22.9	22.7	0.99	63
				90(68)	***200(150)***	***160***	***0.82(1.07)***	13
	ascites*	5.6	27		28.7	29.0	1.01	18
				200(150)	***190(143)***	***165***	***0.88(1.15)***	15
	ascitesF*	3.8	14		17.0	36.0	(~2.1)	56
				82(62)	***135(101)***	***115***	***0.85(1.14)***	34
OC7	plasma*	4.1	20		~21	~32	(~1.5)	76
				230(173)	***250(188)***	***185***	***0.75(0.99)***	11
	ascites*	3.2	16		26.9	28.4	1.05	71
				110(83)	***270(203)***	***230***	***0.85(1.13)***	13
	ascitesF*	4.5	20		22.3	33.4	(~1.5)	75
				120(90)	***140(105)***	***115***	***0.83(1.09)***	13

*analysis of thawed samples; ascitesF = filtered ascites

Table S6. Structural data for ENPs determined by dynamic (DLS) and static light scattering (SLS) measurements for patients with two different benign pathologies (BP) of reproductive organs (myoma of uterus (BP-M) and endometriosis (BP-E)): average hydrodynamic radius for peaks in the size distributions at an angle θ = 90° ($R_{h,90}$), hydrodynamic radius extrapolated to θ = 0° ($R_{h,0}$), radius of gyration (R_g), the shape parameter $\rho(= R_g/R_h)$, and the contribution of exosomes to the total scattering intensity (% $I_{tot,90°}$). For patient BP-M only preliminary DLS measurements at θ = 90° were collected.

		$R_{h,90}$ / nm			angular dependency			
patient	sample	peak 1	peak 2	peak 3	$R_{h,0}$ / nm	R_g / nm	ρ	$\%I_{tot,90°}$
BP-M	plasma	6	23	/				
	PW	9	/	240				
	PW-F	12	/	100				
	PF	6	25	110				
	PF-F	3, 9	24	/				
BP-E	plasma	5	29	120	47.4	48.4	1.0	70
	PW*	7	27	180	39.4	53.9	1.4	44
	PW*-C	5	35		/#	/#	/#	
				260	98	161	1.7	36
	PW*-C+F	8						
				75	56	94	1.7	70
	PF	6	21	165	/#	/#	/#	44
	PF*	5	15	100	22	35	~1.6	40
	PF*-C	10	26	90	/#	/#	/#	/#
	PF*-C+F	11						
				86	62	104	1.7	87

*analysis of thawed sample; F= filtered, C= concentrated by ultracentrifugation, C+F = concentrated by ultracentrifuge and filtered; #analysis of angular dependency was not possible

REFERENCES

1. Maathuis, J.B.; Van Look, P.F.; Michie, E.A. Changes in volume, total protein and ovarian steroid concentrations of peritoneal fluid throughout the human menstrual cycle. *J Endocrinol* **1978**, *76*, 123-133, doi:10.1677/joe.0.0760123.
2. Galic Jerman, K.; Kobal, B.; Jakimovska, M.; Verdenik, I.; Cerne, K. Control values of ovarian cancer tumor markers and standardisation of a protocol for sampling peritoneal fluid and performing washing during laparoscopy. *World J Surg Oncol* **2014**, *12*, 278, doi:10.1186/1477-7819-12-278.
3. Prat, J. Staging classification for cancer of the ovary, fallopian tube, and peritoneum. *Int J Gynaecol Obstet* **2014**, *124*, 1-5, doi:10.1016/j.ijgo.2013.10.001.
4. Urban, C.; Schurtenberger, P. Characterization of Turbid Colloidal Suspensions Using Light Scattering Techniques Combined with Cross-Correlation Methods. *J Colloid Interface Sci* **1998**, *207*, 150-158, doi:10.1006/jcis.1998.5769.
5. Schärtl, W. *Light Scattering from Polymer Solutions and Nanoparticle Dispersions*; Springer: Berlin, Germany, 2007.
6. Brown, W. *Dynamic light scattering: the method and some applications*; Brown, W., Ed.; Clarendon Press: Oxford, England, 1993.
7. Inverse problems. Available online: http://s-provencher.com/index.shtml (accessed on Jan 26).
8. Tarassova, E.; Aseyev, V.; Filippov, A.; Tenhu, H. Structure of poly(vinyl pyrrolidone) – C70 complexes in aqueous solutions. *Polymer* **2007**, *48*, 4503-4510, doi:10.1016/j.polymer.2007.05.069.
9. Božič, D.; Sitar, S.; Junkar, I.; Štukelj, R.; Pajnič, M.; Žagar, E.; Kralj-Iglič, V.; Kogej, K. Viscosity of Plasma as a Key Factor in Assessment of Extracellular Vesicles by Light Scattering. *Cells* **2019**, *8*, 1046, doi:10.3390/cells8091046.
